# A myeloid IFN gamma response gene signature correlates with cancer prognosis

**DOI:** 10.1002/ctm2.70139

**Published:** 2025-03-31

**Authors:** Yuchao Zhang, Asma Khanniche, Yizhe Li, Zhenchuan Wu, Hailong Wang, Hongyu Zhang, Xiaoxue Li, Landian Hu, Xiangyin Kong

**Affiliations:** ^1^ CAS Key Laboratory of Tissue Microenvironment and Tumor Shanghai Institute of Nutrition and Health Chinese Academy of Sciences Shanghai China; ^2^ ANDA Biology Medicine Development (Shenzhen) Co., LTD Shenzhen China; ^3^ Shenzhen Institutes of Advanced Technology Chinese Academy of Sciences Shenzhen China; ^4^ School of Life Science and Technology ShanghaiTech University Shanghai China

**Keywords:** anti‐PD1 immunotherapy, IFN‐γ response genes, LASSO, pan‐cancer, prognosis, tumour microenvironment

## Abstract

**Background:**

The IFN‐γ cytokine plays a dual role in anti‐tumor immunity, enhancing immune defense against cancer cells while promoting tumor survival and progression. Its influence on prognosis and therapeutic responses across cancer types remains unclear.

**Objective:**

This study aimed to perform a pan‐cancer analysis of IFN‐γ response genes to determine their prognostic significance and evaluate their impact on clinical outcomes and anti‐PD1 immunotherapy responses.

**Methods:**

Using multiple datasets, 46 IFN‐γ response genes were identified as prognostic for disease‐specific survival, and their expression was used to construct the IFN‐γ Response Gene Network Signature (IFGRNS) score. The prognostic and therapeutic relevance of the IFGRNS score was assessed across cancer types, considering tumor pathology, genomic alterations, tumor mutation burden, and microenvironment. Single‐cell transcriptomic analysis identified cellular contributors, and a murine pancreatic cancer (PAN02) model was used to validate findings with anti‐PD1 therapy.

**Results:**

The IFGRNS score emerged as a robust prognostic indicator of survival, with higher scores correlating with worse outcomes in most cancer types. The prognostic significance of the score was influenced by factors such as cancer type, tumor pathology, and the tumor microenvironment. Single‐cell analysis revealed that myeloid cells, particularly the M2 macrophage subtype, demonstrated high levels of IFGRNS expression, which was associated with tumor progression. A negative correlation was observed between the IFGRNS score and outcomes to anti‐PD1 immunotherapy in urologic cancers, where patients with higher scores showed worse prognosis and lower response rates to therapy. Experimental validation in the PAN02 murine model confirmed that anti‐PD1 therapy significantly reduced tumor size and IFGRNS expression in M2 macrophages, supporting the clinical findings.

**Conclusions:**

The IFGRNS score is a novel prognostic indicator for survival and therapeutic responses in cancer. These findings underline the complexity of IFN‐γ signaling and suggest potential applications for the IFGRNS score in cancer diagnosis, prognosis, and immunotherapy.

**Novelty & impact statements**: IFN‐γ response genes play a significant role in tumour biology, yet comprehensive analysis across various cancers is limited. This study identifies a novel prognostic biomarker, the IFGRNS score, which is elevated in myeloid lineage cells and correlates with survival across multiple cancers. The IFGRNS score is also associated with tumour pathology, immune microenvironment, and immunotherapy response, highlighting its diagnostic and therapeutic potential in cancer management.

**Key points:**

- IFN‐γ cytokine plays a dual role in cancer, aiding immune defense but also promoting tumor progression.
- A novel IFGRNS score, based on 46 IFN‐γ response genes, was identified as a strong prognostic marker for survival across cancer types.
- Higher IFGRNS scores correlate with worse prognosis and reduced response to anti‐PD1 immunotherapy, particularly in urologic cancers.
- M2 macrophages were identified as key contributors to high IFGRNS scores, associated with tumor progression.
- Findings were validated in a murine cancer model, highlighting the potential of the IFGRNS score for cancer prognosis and therapy guidance.

## Graphical abstract

1. IFGRNS score is a novel prognostic biomarker upregulated in myeloid lineage cells.
2. IFGRNS score correlates with survival outcomes across multiple cancer types.
3. IFGRNS score is associated with tumour pathology and immune microenvironment.
4. IFGRNS score predicts immunotherapy response, with potential for clinical application.

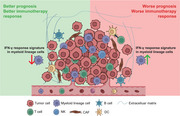

## 1 INTRODUCTION

Interferon‐gamma (IFN‐γ) is a critical cytokine in tumour immunology, playing a dual role in both promoting anti‐tumour immunity and, paradoxically, contributing to tumour progression under certain conditions.^1^ On the one hand, IFN‐γ enhances immune responses with cytostatic, pro‐apoptotic, and anti‐proliferative effects, making it a potential adjuvant in immunotherapy.^2^ It inhibits tumour angiogenesis, induces apoptosis of regulatory T cells, and activates M1 macrophages, all of which can suppress tumour growth.^3^, ^4^ However, under specific conditions, IFN‐γ signalling can also drive tumour survival, metastasis, and resistance to immune checkpoint blockade (ICB) by mechanisms such as downregulation of major histocompatibility complex (MHC) molecules, increased expression of indoleamine 2,3‐dioxygenase (IDO), and upregulation of PD‐L1.^2^, ^5^, ^6^, ^7^

Persistent IFN‐γ signalling, particularly through interferon‐stimulated genes (ISGs), may contribute to immune evasion and therapy resistance.^8^, ^9^, ^10^, ^11^ For example, Joseph et al.^8^ demonstrated that high levels of ISG expression in tumours, driven by chronic IFN‐γ signalling, are associated with resistance to ICB, highlighting the context‐dependent role of IFN‐γ in cancer.

Although previous studies have explored the role of IFN‐γ and its related genes in various cancers,^12^, ^13^, ^14^, ^15^ including their involvement in proliferation, apoptosis, metastasis, and immune responses, the findings across different cancer types have been inconsistent. This reflects the complexity of the tumour microenvironment and the immune system.^16^, ^17^, ^18^

IFN‐γ response genes, which are regulated by the IFN‐γ/IFNGR/JAK/STAT1 signalling pathway, are involved in key biological processes, including antigen presentation, inflammation, and immune responses.^2^, ^12^, ^19^ However, their clinical significance, particularly in predicting cancer prognosis and response to immunotherapy, remains unclear.

In this study, we aim to address this knowledge gap by developing a pan‐cancer model based on IFN‐γ response genes. Using the Least Absolute Shrinkage and Selection Operator (LASSO) Cox model, we established an IFN‐γ response signature score (IFGRNS score) and evaluated its correlation with gene mutations, clinical features, tumour microenvironment, and immunotherapy response. We demonstrate that the IFGRNS score serves as a novel prognostic indicator across multiple cancer types, offering potential applications in predicting outcomes and guiding treatment strategies.

## 2 MATERIALS AND METHODS

### 2.1 Datasets

In this study, we collected and analyzed pan‐cancer data from The Cancer Genome Atlas program (TCGA). The TCGA data sets, which were obtained from the USCS Xena hub (http://xenabroswer.net/hub), comprised 8980 cases of pan‐cancer from 32 types of tumours. The clinical information of patients from the TCGA database was downloaded from the cbioportal database (https://www.cbioportal.org), which provided structured and pre‐processed clinical data including cancer type, stage, mutation count, overall survival (OS), disease‐specific survival (DSS), and progression‐free interval (PFI). Each patient in the TCGA dataset has a unique Patient ID, allowing for precise matching of clinical data with gene expression data. To develop and validate an IFGRNS‐based prognostic model, we divided the TCGA data into a training set (70%) and a test set (30%) randomly. However, some cancer types in the TCGA data had insufficient events (less than 10) in either the training or the testing set, which would compromise the reliability of the survival analysis. Therefore, we excluded these datasets (TCGA‐CHOL, TCGA‐DLBC, TCGA‐KICH, TCGA‐PCPG, TCGA‐PRAD, TCGA‐READ, TCGA‐TGCT, TCGA‐THCA, and TCGA‐THYM) from survival analysis.

To further validate the IFGRNS‐based model, we collected gene expression and matched survival data from 14 independent external datasets including the Chinese Glioma Genome Atlas (CGGA, *n* = 1314), the Molecular Taxonomy of Breast Cancer International Consortium (METABRIC, *n* = 1980), and other datasets from the GEO database, which consisted of patients with breast cancer (GSE12276 [*n* = 195], GSE17705 [*n* = 298], GSE19615 [*n* = 115], GSE21653 [*n* = 252], GSE2990 [*n* = 122], GSE7390 [*n* = 198]), pancreatic ductal adenocarcinoma (PDAC, GSE183795 [*n* = 134]), lung cancer (GSE19188 [*n* = 82], GSE3141 [*n* = 111], GSE8894 [*n* = 138], GSE43767 [*n* = 69]), and rectal/colorectal cancer (GSE40492 [*n* = 219], GSE87211 [*n* = 196], GSE39582 [*n* = 574], GSE33113 [*n* = 89], GSE14333 [*n* = 226]).

Concerning the expression and the survival data (or tumour response assessment) from the anti‐PD1 immunotherapy clinical trial cohorts, we included nine datasets: IMmotion150 (a phase II trial in untreated metastatic renal cell carcinoma [mRCC] patients with atezolizumab [Atezo] and/or bevacizumab [Bev], *n* = 263), IMvigor210 (a phase II trial in metastatic urothelial cancer [mUC] patients with Atezo, *n* = 354), POPLAR (a phase II trial in non‐small‐cell lung cancer patients with Atezo, *n* = 193), GSE140901 (Hsu et al., advanced/metastatic hepatocellular carcinoma patients with anti‐PD‐1/anti‐PD‐L1 therapy, *n* = 24), JAVELIN Renal 101 (first‐line avelumab plus axitinib in patients with advanced renal cell carcinoma, *n* = 886), GSE136961 (Sohyun Hwang et al., nivolumab or pembrolizumab in non‐small‐cell lung cancer patients, *n* = 21), GSE78220 (Hugo et al., nivolumab or pembrolizumab in melanoma patients, *n* = 39), CA209‐038 study (Riaz et al., Nivolumab in patients with advanced melanoma including tumour samples at pre‐treatment and on‐treatment, *n* = 58), and GSE176307 (Rose et al., Atezolizumab or Pembrolizumab or Nivolumab or Durvalumab or Avelumab in mUC patients, *n* = 89).

### 2.2 LASSO Cox regression model

We applied the LASSO algorithm to select the most relevant prognostic genes for IFN‐γ response genes from a large pool of candidates. LASSO regression was performed by the R package ‘glmnet’ (v4.1.7),^20^ with the parameter ‘family = cox’. The optimal regularization parameter (λ) was chosen based on the one‐standard‐error rule from cross‐validation. This approach helped prevent overfitting and identified the most parsimonious model. To address multicollinearity among genes, LASSO inherently penalizes large coefficients, effectively handling correlated features by shrinking some to zero and selecting a subset of non‐zero coefficient genes.

### 2.3 IFGRNS scores

We used the univariate Cox proportional hazard regression model to further select the genes that were significantly correlated with DSS. Then, we used multivariate Cox regression to evaluate how the expression levels of each gene were associated with DSS and obtained each coefficient β of those genes. Cox regression was performed by function ‘coxph’ of R package ‘survival’ (v3.5.5).^21^ We calculated the IFGRNS score for each patient based on the coefficient β. The IFGRNS score was a weighted sum of the normalized expression values of the genes in the IFGRNS signature, where the weights were the regression coefficient βs of the genes in the multivariate Cox regression analysis. The IFGRNS score represented the risk level of each patient for DSS. We used the median IFGRNS score as a threshold to classify the tumour samples into high‐score and low‐score groups. The median IFGRNS score varies between tumour types due to differences in gene expression profiles and tumour biology across cancers. The prognostic value of the IFGRNS score and its application to predict survival outcomes may require dataset‐specific thresholds.

### 2.4 Evaluation of the immune microenvironment, inflammation, and responsive/resistance to anti‐PD‐1 immunotherapy signatures

The gene sets of known signatures of immune, inflammation, and anti‐PD‐1 immunotherapy were obtained from the published literature (immune cells were from Bindea et al.,^22^ inflammation and TGF‐β‐associated ECM were from Chakravarthy et al.,^23^ activated stroma were from Moffitt et al.,^24^ Nivolumab responsive were from Riaz et al.,^25^ anti‐PD‐1 resistant were from Hugo et al.,^26^ Ayers 6‐gene IFN‐γ signature or expanded immune signature were from Ayers et al.,^14^ Pan‐fibroblast TGFβ signature were from Mariathasan et al.,^27^ tumour immune dysfunction and exclusion (TIDE) signature score were download from Peng et al.,^28^ and IFN‐stimulated genes resistance signature (ISG.RS) and its complementary subset IFNG.GS were from Joseph et al.^29^). We computed single‐sample gene set enrichment analysis (ssGSEA) scores using those gene sets for the signatures. ssGSEA was performed using the R package ‘GSVA’ (v1.46.0).^30^ To calculate the innate anti‐PD‐1 resistance (IPRES) scores, we followed the method of Hugo et al.,^26^ who defined IPRES based on 26 gene sets. IPRES was calculated as the average Z‐score across all gene sets.

### 2.5 Murine lung cancer model

A lung cancer model was established in LSL‐KrasG12D mice, which were obtained from Shanghai Model Organisms Center, Inc. The model was induced through the intranasal administration of adenovirus‐Cre recombinase (AD‐Cre) at a concentration of 2 × 10^6^ PFU. Experimental approval was granted by the Institutional Animal Care and Use Committee of the Shanghai Institute of Nutrition and Health, CAS, and the guidelines for animal experiment management (SINH‐2020‐KXY‐1) were strictly adhered to. Mice were euthanized at 2‐, 6‐, 12‐, and 20 weeks postadministration, corresponding to critical stages in the progression of lung adenocarcinoma (LUAD).

### 2.6 Murine pancreatic cancer model and anti‐PD‐1 treatment

Murine pancreatic cancer PAN02 cells (purchased from Shanghai Zishi Biotechnology Co., Ltd.) were preserved in our laboratory and cultured in DMEM medium supplemented with 10% FBS at 37°C with 5% CO_2_. Cells were collected for inoculation when cells were 80–90% confluent. A total of 1.8 × 10^6^ PAN02 cells in .1 mL suspension were inoculated subcutaneously into the right lower flank of each mouse. Once tumours reached an average volume of 50 mm^3^ (approximately 7–10 days post‐inoculation), mice were randomly divided into two groups (8 mice/group), and the treatment was initiated. The date of randomization is designated as Day 0. Starting on Day 1, the treatment group received 100 µL (200 µg/mouse) of InVivoMAB anti‐mouse PD‐1 (RMP1‐14, BioXcell) via intraperitoneal injection on Days 1, 3, 5, 7, 9, 16, 23, and 31, for a total of eight doses over the course of 31 days. The vehicle control group was administered an equal volume of NaCl following the same schedule. The diameter of the tumours and the body weight of the mice were measured twice a week. The formula for calculating tumour volume (TV) is the following: TV = 1/2 × *a* × *b* ^2^, where *a* represents the tumour length and *b* represents the tumour width. Tumour volume and body weight data were collected and analyzed to assess the impact of anti‐PD‐1 treatment. Statistical analyses were conducted using GraphPad Prism V.7. Differences among multiple groups were calculated by two‐way ANOVA or otherwise indicated, with *p* ≤ .05 considered a significant difference.

The preparation of this protocol and any modifications has been approved by the Experimental Animal Management and Use Committee (IACUC) of the Shanghai Institute of Materia Medica, Chinese Academy of Sciences. The use and welfare of laboratory animals were carried out in accordance with the regulations of the International Commission for the Evaluation and Accreditation of Experimental Animals (AAALAC). The health of the animals was monitored daily, and routine examinations of tumour growth, behaviour, activity, water intake, weight changes, and physical appearance were conducted twice weekly. Any deaths or side effects were recorded throughout the study.

### 2.7 Murine sample collection and processing for single‐cell RNA sequencing

For the murine lung cancer model, whole lung tissues from 2‐, 6‐, 12‐, and 20 weeks post‐administration were collected. Single‐cell suspensions were prepared from whole lung tissue using the lung dissociation kit and tumor dissociation kit provided by Miltenyi Biotech. Cells were then separated into CD45^+^ and CD45^−^ populations using microbeads and filtered through a 35 µm strainer. The resulting single cells were counted to generate a high‐quality single‐cell library. The single‐cell library was constructed using the 10X Genomics Chromium Next GEM Single Cell 3′ GEM Library & Gel Bead Kit v3.1 for CD45^−^ cells and the Chromium Next GEM Single Cell 5′ Library & Gel Bead Kit v1.1 for CD45^+^ cells, according to the manufacturer's instructions. Sequencing was performed on the Illumina NovaSeq PE150.

For anti‐PD1 therapy in the murine pancreatic cancer model, we randomly selected 1 mouse from each group and collected peripheral blood and tumour tissues for single‐cell sequencing. Peripheral blood samples were obtained from mouse eye sockets. After the collection of blood, each sample was first mixed with 200 µL EDTA, followed by mixing with an equal volume of PBS. Equal volumes of Ficoll Paque PREMIUM (Amersham/GE) and blood‐EDTA‐PBS solution were added to 15 mL centrifuge tubes and centrifuged at 400×*g* for 20 min to collect peripheral blood single nucleated cells (PBMCs), after which erythrocytes were removed with erythrocyte lysis solution (ThermoFisher) to remove erythrocytes. The remaining cells were filtered through a 30 µm (Miltenyi/MACS) filter, washed 1–2 times with PBS, and resuspended in PBS to the appropriate volume. Cells were subsequently stained with AO/PI (Nexcelom Bioscience) and quantified in vivo using Cellometer K2 (Nexcelom Bioscience). Tumour tissues were collected and cut into small pieces (approximately 1–2 mm). Afterwards, the tumour tissues were digested using the Mouse tumour tissue dissociation kit (Miltenyi/MACS) according to standard procedures and the cell suspension was filtered through a 30 µm filter (Miltenyi/MACS). After the cell suspension was washed with PBS, the cells were also stained with AO/PI and quantified in vivo using Cellometer K2 (Nexcelom Bioscience). Final mouse PBMC or tumour cell samples were obtained for library construction using the 10X Genomics standard process. The library building kit includes Chromium Next GEM Single Cell 3′ GEM, Library & Gel Bead Kit v3.1, Chromium Next GEM Chip G Single Cell Kit, and Single Index Kit T Set A. The experimental procedure was carried out according to the manufacturer's instructions. Follow‐up sequencing was performed by Novozymes using the Illumina NovaSeq PE150 platform.

### 2.8 Single‐cell RNA‐seq data processing

After we got the mouse single‐cell sequencing data, we first analyzed each sample, initially, using the CellRanger (v3.1.0) standard process. Sequencing reads were reads mapped to the mm10 mouse reference sequence. After obtaining the expression matrix, the R package Seurat was employed for data analysis. The R package Harmony (v0.1.1) was used to mitigate batch effects during data aggregation. After aggregating all samples, the number of genes was quantified to be between 200 and 3500, and mitochondrial gene content was maintained at less than 15%. Aggregated data were normalized, the top highly variable genes were selected, total gene and mitochondrial reads were regressed out, a principal component analysis was performed, and the first 50 principal components were used for nearest neighbour calculations and Leiden clustering (resolution = 1), as well as for uniform manifold approximation and projection‐based visualization. Cell annotation was performed using immune cell type markers and highly specific expressed genes. After an initial, global analysis, macrophages, monocyte/DC cells, B cells, T cells, and tumour cells were reanalyzed and annotated separately, and the final annotation was obtained from manual integration of all analyses. As IFGRNS was based on the bulk RNA‐seq, we used pseudo‐bulk analysis for the assessment of IFGRNS across cell types or subtypes in single‐cell sequencing. We summed expression values across all cells belonging to a sample across subtypes of cells and then obtained the pseudo‐bulk data, which was employed to estimate the IFGRNS scores as described above.

### 2.9 Statistical analysis

To avoid the influence of different scales and units, we standardized the training and test sets using zero‐mean normalization, which transforms the data to have a mean of zero and a standard deviation of one. We used the Kaplan−Meier analysis to estimate the differences in survival in patients within low‐risk and high‐risk groups and draw the survival curves. The patients in each cohort were divided into high‐score and low‐score groups based on the cutoff value of the IFGRNS. For the TCGA cohorts, we determined the cut‐off based on the median value of IFGRNS scores. For external datasets, we identified the optimal cut‐off using the cutp function from the SurvMisc package (version 0.5.6), which selects the best threshold based on survival outcomes. To mitigate the bias and overfitting introduced by this method, *p*‐values were adjusted for multiple testing across various cut points. We compared the survival curves of the two groups using the log‐rank test, which tests the null hypothesis that there is no difference in survival between the groups. The effects of the IFGRNS score on the patients’ survival were evaluated as a hazard ratio (HR) and 95% CI that were derived from the Cox proportional hazard regression. We also measured the prediction performance of the outcomes of 3, 5, and 10‐year survival by the areas under the curves (AUC) of the IFGRNS score using the time‐dependent receiver operating characteristic curve, which is a plot that shows the trade‐off between sensitivity and specificity at different time points. Comparisons of IFGRNS scores between multiple groups in categorical clinical characteristics were performed using the Kruskal test, comparisons between two groups were performed using the Wilcox test, and differences in IFGRNS scores were demonstrated using a boxplot. Correlations between numerical clinical characteristics or immune signatures and IFGRNS scores were tested using the Spearman correlation test, and correlations were expressed as correlation coefficients and *p*‐values. The correlations between the IFGRNS score and somatic mutations in genes were tested using the point‐biserial correlation method. To construct and validate a nomogram, which is a graphical representation of a predictive model that shows the relationship between the predictors and the outcome, we performed multivariable Cox regression analysis, which models the hazard rate as a function of covariates. The nomogram integrated multiple forecast indicators to provide a personalized prediction of survival for each patient. We evaluated the clinical benefit provided by model of IFGRNS score plus clinical features using decision curve analysis. All reported *p*‐values are two‐sided, and the significance level was set at .05 for all analyses unless otherwise noted. The R/Bioconductor programming environment (R, version 3.6.1) was used for all statistical analyses and plotting.

## 3 RESULTS

### 3.1 IFN‐γ response (IFGRNS) score is associated with prognosis in multiple cancer types

**FIGURE 1:**
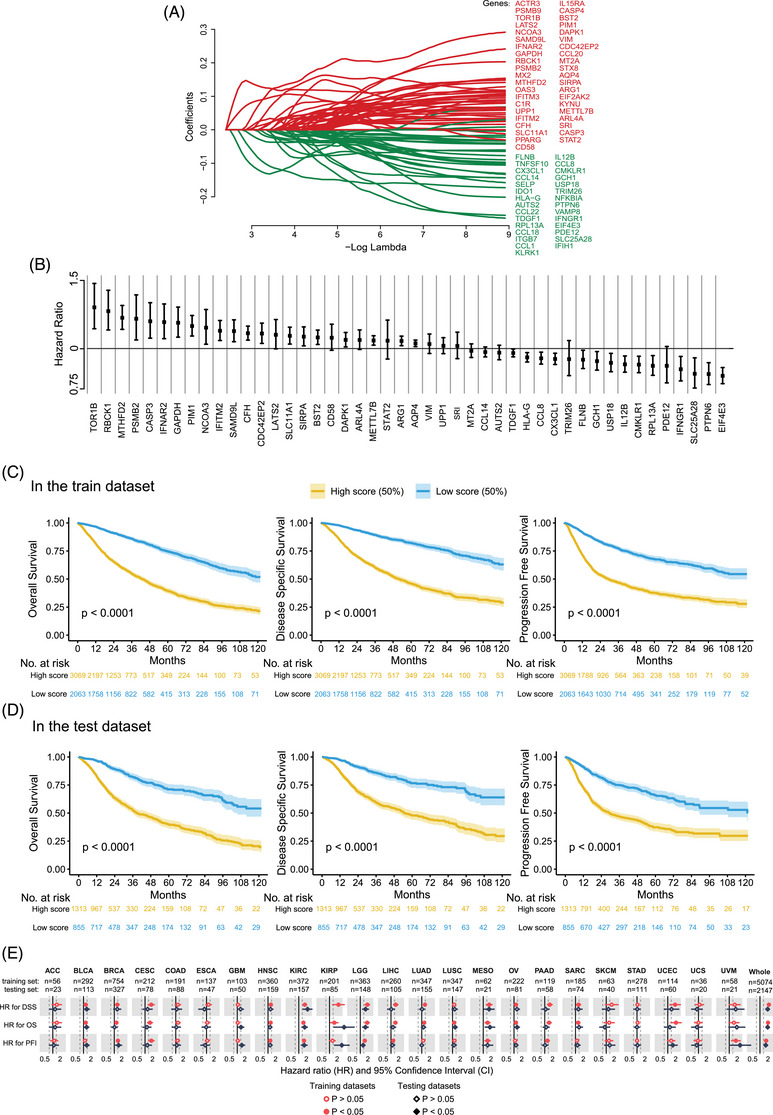
Construction of the IFN‐γ response gene‐based model that is prognostic for disease‐specific survival. (A) 70 genes were selected using LASSO Cox regression analysis for disease‐specific survival. The coefficient profiles of those genes were shown. (B) A forest plot showing the point estimates and confidence intervals of the HRs for each of those 46 genes derived from the result of the multivariate Cox regression analysis. The squares indicate the HRs and the whiskers indicate the confidence intervals of HRs. (C, D) Kaplan−Meier survival curves of patients with high IFGRNS scores and low IFGRNS scores, in the train dataset (C) and in the test dataset (D). (E) Hazard ratio (HR) and 95% confidence interval (CI) for disease‐specific survival (DSS) of the IFGRNS scores for the training sets (red) and testing sets (black) in the cohorts of TCGA data. The patients in each dataset were divided into high‐score and low‐score groups by the median of the scores. Hazard ratios were calculated with COX regression (high‐score group vs. low‐score group).

To assess the association between IFN‐γ response genes and survival outcomes of patients with primary tumours, we applied the LASSO Cox model to construct IFGRNS scores for the pan‐cancer dataset (Figure S1). We obtained 316 IFN‐γ response‐related genes from the Gene Set Enrichment Analysis (GSEA) database under the entries “HALLMARK INTERFERON GAMMA RESPONSE” and “GOBP RESPONSE TO TYPE II INTERFERON”. We then used these genes for LASSO regression analysis. The data used were from TCGA pan‐cancer data comprising transcriptome data of 32 types of solid primary tumours (refer to Table S1 for abbreviations and full names of cancers) and survival data of 8980 patients. Survival data included OS, DSS, and tumour PFI. We split the TCGA pan‐cancer data into a training set and a test set at a ratio of 7:3. We performed LASSO regression analysis only on the training set, analyzing the IFN‐γ response‐related genes related to DSS, and identified 70 genes with non‐zero coefficients from 316 genes (Figure 1A). Then, considering that these genes may have interdependencies among themselves, we performed pairwise correlation analysis on these 70 genes and eliminated 19 highly correlated genes (Figure S2). From the remaining 51 genes, further univariate COX regression analysis selected 46 genes that were significantly associated with the outcomes of DSS. Subsequently, the model construction used multivariate COX regression analysis to examine the relationship between those 46 genes and patient DSS and obtain the coefficients of each gene (Figure 1B). The sum of the product of the coefficient and the normalized gene expression value of each gene was calculated for each tumour sample, and it was defined as the IFGRNS score.

To verify the correlation between the IFGRNS score and the survival outcomes of patients, we stratified all patients into high and low‐score groups based on the median value of the IFGRNS score and plotted the Kaplan−Meier curves of the high and low groups (Figure 1C,D). The results indicated that both in the training set and in the test set, patients with high IFGRNS scores had significantly worse outcomes of DSS, OS, and PFI compared with the low‐score group. This confirmed the reliability of this IFGRNS‐based risk model. Similarly, we also compared the Kaplan‐Meier curves of high and low‐score groups in each of the 32 cancer types (Figures S3–S8). We found that the IFGRNS score was significantly associated with survival outcomes in 10 cancer types (HNSC, KIRC, KIRP, LGG, MESO, SARC, BLCA, LIHC, LUAD, and UVM) in both the training set and test set. Those results revealed that the high score group of IFGRNS was linked to poor prognosis in solid tumours (Figure 1E and Table 1 for the HR and *p*‐value based on the COX regression analysis). Moreover, the IFGRNS score as a predictor could effectively predict 3‐year survival, 5‐year survival, or 10‐year survival of seven types of cancer (UVM, UCEC, PAAD, MESO, LGG, KIRP, and KIRC). However, IFGRNS scores had lower predictive accuracy for 3‐ and 5‐year survival for PAAD, 10‐year survival for mesothelioma (MESO), and 10‐year survival for low‐grade glioma (LGG). These results suggest that the IFGRNS score reflected a negative prognostic association.

**TABLE 1:** Significant correlation between IFGRNS scores and outcomes of DSS, OS, and PFI in each type of cancer in train and test datasets.^a^

		IFGRNS scores in train dataset				IFGRNS scores in test dataset			
Survival	Cancer	HR	95% CI lower	95% CI upper	*p*‐value	HR	95% CI lower	95% CI upper	*p*‐value
DSS	HNSC	1.72	1.3	2.27	<.001	1.632	1.073	2.481	.022
DSS	KIRC	2.84	2.15	3.74	<.001	3.321	2.139	5.154	<.001
DSS	KIRP	4.26	2.76	6.57	<.001	10.263	2.949	35.715	<.001
DSS	LGG	3.72	2.88	4.81	<.001	2.493	1.733	3.587	<.001
DSS	MESO	3.36	1.97	5.75	<.001	2.194	1.021	4.715	.039
DSS	SARC	1.59	1.14	2.22	.007	2.048	1.147	3.659	.015
DSS	Whole	2.75	2.6	2.92	<.001	2.525	2.31	2.76	<.001
OS	BLCA	1.59	1.21	2.08	.001	1.463	1.006	2.128	.047
OS	HNSC	1.57	1.24	2	<.001	1.559	1.099	2.21	.012
OS	KIRC	2.25	1.78	2.86	<.001	2.426	1.697	3.468	<.001
OS	KIRP	3.42	2.27	5.16	<.001	6.909	2.405	19.846	<.001
OS	LGG	3.65	2.83	4.7	<.001	2.417	1.691	3.455	<.001
OS	LIHC	2.49	1.74	3.56	<.001	1.562	1.003	2.435	.048
OS	LUAD	1.96	1.47	2.63	<.001	1.988	1.214	3.257	.006
OS	MESO	2.36	1.44	3.88	.001	2.596	1.258	5.358	.008
OS	SARC	1.41	1.02	1.95	.036	2.022	1.192	3.43	.009
OS	Whole	2.47	2.35	2.6	<.001	2.41	2.229	2.606	<.001
PFI	BLCA	1.58	1.21	2.06	.001	1.494	1.004	2.222	.048
PFI	KIRC	2.52	1.97	3.22	<.001	2.517	1.712	3.699	<.001
PFI	KIRP	2.76	1.94	3.94	<.001	5.671	2.375	13.54	<.001
PFI	LGG	2.64	2.17	3.22	<.001	1.921	1.426	2.588	<.001
PFI	UVM	7.58	2.75	20.87	<.001	18.456	1.937	175.836	.011
PFI	Whole	2.11	2.01	2.21	<.001	2.026	1.885	2.177	<.001

Abbreviations: DSS, disease‐specific survival; OS, overall survival; PFI, tumour progression‐free interval.

^a^ HR and *p*‐value were obtained from the Cox regression analysis. It only shows the results that were both significant (*p* < .05) in the train set and test set.

### 3.2 IFGRNS score is associated with cancer prognosis in independent external datasets

**FIGURE 2:**
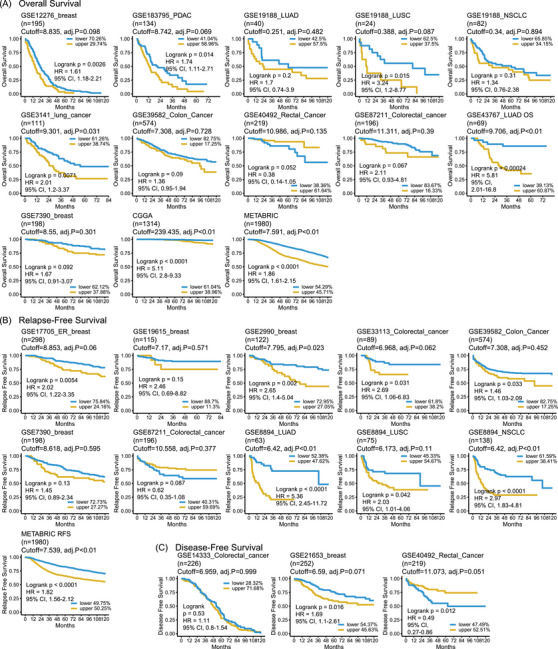
The prognostic value of the IFGRNS score in different types of cancer in external datasets. Kaplan−Meier survival curves of patients with high and low IFGRNS scores in the external validated datasets, including patients with glioma, breast cancer, pancreatic ductal adenocarcinoma, lung cancer, rectal cancer, and colorectal cancer. (A) Overall survival (OS) of patients stratified by the IFGRNS score. (B) Tumour relapse‐free survival (RFS) of patients stratified by the IFGRNS score. (C) Disease‐free survival (DFS) of patients stratified by the IFGRNS score. The patients in each dataset were divided into high‐score and low‐score groups based on the optimal cutoff value of the IFGRNS score, which was determined by the ‘cutp’ function in the ‘survMisc’ package for R. The size of population (percent of patients) for high‐score and low‐score groups are labelled in the graph. The optimal cutoff value and the adjusted p value for each dataset are shown above the graph. The adjusted *p*‐value was calculated by correcting for multiple testing across different cutoff values using the method of Contal and O'Quigley. NSCLC, non‐small‐cell lung cancer.

Next, we confirmed the correlation between IFGRNS scores and survival outcomes using external datasets including breast cancer, pancreatic cancer, lung cancer, colorectal cancer, and glioma cohorts. IFGRNS scores were linked to worse survival in breast, lung, pancreatic, and gliomas (Figure 2). These results were consistent with those of the TCGA data analysis suggesting that the score model was a robust and reliable prognostic biomarker.

It is noteworthy that the IFGRNS score was associated with survival in a heterogeneous manner across the datasets of colorectal cancers. Specifically, a high IFGRNS score was correlated with poor survival in GSE39582 (colon cancer, *n* = 574), and GSE33113 (colorectal cancer, *n* = 89), which was consistent with our conclusion. However, we also observed that a high IFGRNS correlated with better survival in GSE40492 (rectal cancer, *n* = 219) and GSE87211 (colorectal cancer, *n* = 196), which was contrary to the effect of IFGRNS in TCGA data, although IFGRNS score was not significantly associated with survival in colorectal cancers in TCGA (*p* = .08 in the training set and *p* = .2 in the testing set).

To explore these discrepancies, we considered differences in tumour microenvironment (TME) composition and immune infiltration across cohorts. Subgroup analyses revealed that datasets with significantly less CD8+ T cell, cytotoxic immune cell, and Th2 cell infiltration (e.g., GSE33113) were associated with worse outcomes, whereas datasets with significantly more macrophages (e.g., GSE40492 and GSE87211) correlated with better outcomes (Figure S9A,B). We expanded the details of differential gene expression analysis between the high and low IFGRNS groups in each dataset. Genes that were significantly differentially expressed (false discovery rate < .05, log2 fold change > 1) were identified, and pathway enrichment analysis was performed using GSEA to reveal key biological processes driving these discrepancies. This analysis revealed that in GSE40492 and GSE87211, the high IFGRNS group had upregulated pathways related to cytokine‐mediated signalling and cell chemotaxis (Figure S9C–E), suggesting enhanced immune response and inflammation, which may have tumour‐suppressive effects.^31^ These findings suggest that variations in immune cell infiltration and TME composition play critical roles in modulating the prognostic value of IFGRNS across different datasets.

### 3.3 IFGRNS score correlates with tumour pathology and genomic alterations in cancers

**FIGURE 3:**
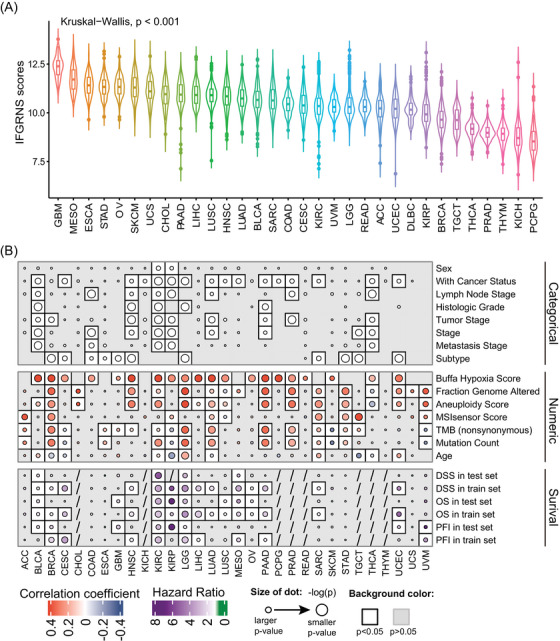
The correlations between the IFGRNS score and clinical characteristics in each cancer type. (A) The violin plot showing the IFGRNS scores across the cancer types. (B) A heatmap showing the correlations of IFGRNS score to the clinical characteristics and genomic alterations in each cancer type. The top panel consists of the categorical features and the middle panel consists of the numeric features. The bottom panel consists of the correlation with prognosis (OS, DSS, and PFI) in training and testing datasets, respectively. The size of the dot indicates the *p*‐values. The filled colour of the dot indicates the correlation coefficient or hazard ratio for survival. The white colour of the background indicates the significance (*p* < .05) of the correlation.

We then examined the correlation between IFGRNS scores and clinical baseline characteristics and genomic alteration features of patients. As expected, the IFGRNS scores differed significantly based on cancer types (Figure 3A). Patients with poor prognoses (e.g., GBM) had the highest IFGRNS scores, while those with relatively favourable prognoses (e.g., PCPG) had the lowest scores. Moreover, IFGRNS scores were significantly correlated with pathology stages (histologic grade [G], size of the primary tumour [T], nodal status [N], and distant metastasis [M], according to the criteria from the American Joint Committee on Cancer [AJCC]), genders, and ages. IFGRNS scores were also significantly correlated with genomic alteration features such as the aneuploidy scores, fraction genome altered scores, microsatellite instability sensor score, mutation count, tumour mutation burden (TMB), and Buffa Hypoxia score. Further stratified analysis by cancer type (Figure 3B) revealed that in most solid tumours, IFGRNS scores were significantly correlated with person neoplasm cancer status (with tumour or tumour free), and they showed significant positive correlations with aneuploidy score, fraction genome altered and Buffa Hypoxia scores. IFGRNS scores were also significantly correlated with pathology stages and tumour subtypes in many solid tumours (Figures S10 and S11). The correlation of IFGRNS scores with TMB varied across cancers. In LGG, LUAD, BRCA, ACC, STAD, and PAAD, IFGRNS scores had significant positive correlations with TMB, but in CESC, KIRP, SKCM, and UVM, they had significant negative correlations with TMB. However, IFGRNS tended to be significantly positively correlated with TMB or mutation count in cancers where IFGRNS was associated with DSS or PFI in both training and testing datasets (Figure 3B). Interestingly, the correlation between IFGRNS scores and TMB was inconsistent between the three subtypes of kidney cancer (KICH, KIRC, and KIRP), and between the two subtypes of lung cancer (LUAD and LUSC).

### 3.4 Establishment of a comprehensive model that combined the IFGRNS score and clinical characteristics

**FIGURE 4:**
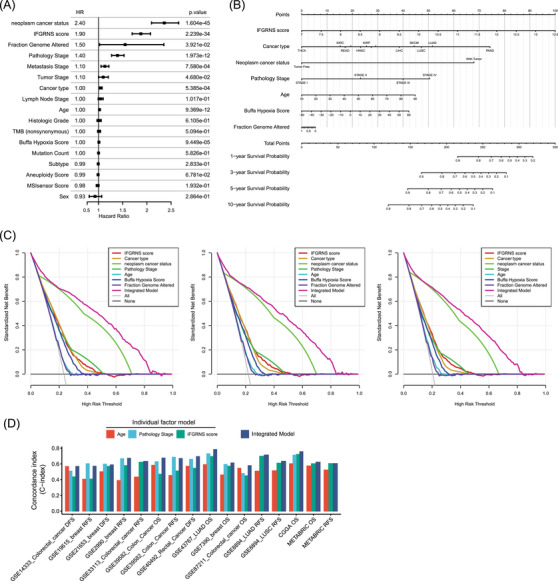
Construction of a comprehensive model that combined the IFGRNS score and clinical characteristics. (A) A forest plot showing the point estimates and confidence intervals of the HRs for each of risk factors derived from the result of the multivariate Cox regression analysis. The squares indicate the HRs and the whiskers indicate the confidence intervals of HRs. (B) A nomogram including IFGRNS score, person neoplasm cancer status, fraction genome altered, cancer type, pathology stage, age, and Buffa Hypoxia score, for 1‐, 3‐, 5‐ and 10‐year disease‐specific survival. (C) Decision curve analysis for the 3, 5, or 10‐year DSS outcomes, respectively. Black line: All patients had the events. Gray line: None patients had the events. Magenta line: the integrated model of nomogram. (D) Comparison of sensitivity and specificity between the integrated model and the individual models in external datasets. The concordance index (C‐index) is a measure of how well the model discriminates between patients with different survival outcomes. It is similar to the area under the ROC curve (AUC), but it can handle censored data. A higher C‐index means that the model can more accurately rank the survival times according to the risk scores.

Given the significant correlation between IFGRNS scores and various clinical factors, we further explored how the combination of IFGRNS scores and these factors affected the DSS of patients using multivariate COX regression analysis (Figure 4A). The analysis revealed that the IFGRNS score remained a strong predictor of DSS, suggesting that IFGRNS can serve as an independent prognostic factor. Moreover, other variables such as person neoplasm cancer status, fraction genome altered, cancer type, pathology stage, age, and Buffa Hypoxia score also showed significant associations with DSS in the multivariate model.

To translate these findings into a practical and user‐friendly tool for clinical use, we developed a comprehensive nomogram that integrated the IFGRNS score and some relevant pathological characteristics, namely person neoplasm cancer status, fraction genome altered, cancer type, pathology stage, age, and Buffa Hypoxia score (Figure 4B). We excluded the metastasis stage from the integrated model because it is part of the pathological stage, which is more significant and avoids multicollinearity. The nomogram demonstrated superior predictive performance for 3, 5, or 10‐year DSS outcomes (Figure 4C). To further validate this integrated model, we applied it to independent external datasets comprising different types of cancer. We observed that the integrated model outperformed the individual models in terms of sensitivity and specificity in most of the external datasets (Figure 4D), suggesting that this model that combined IFGRNS with clinical characteristics was robust and consistent across various data sources.

### 3.5 IFGRNS signature is enriched in myeloid cell lineage and correlates with the tumour microenvironment

**FIGURE 5:**
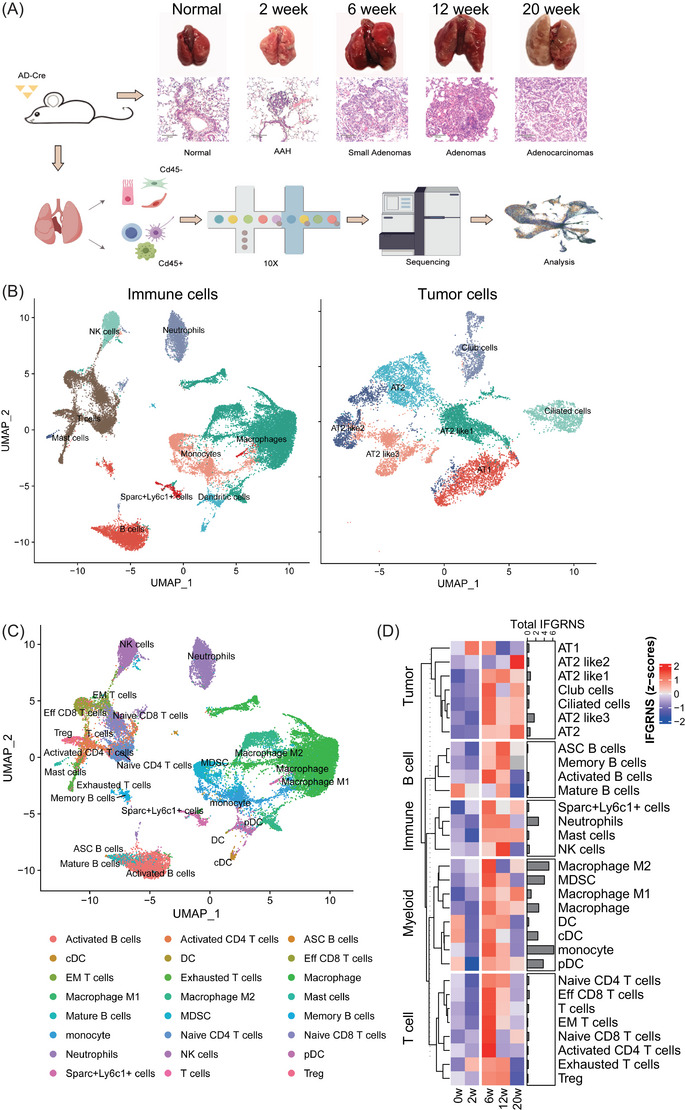
The correlations of the IFGRNS score to the signatures of the tumour immune microenvironment. (A) The design of the lung cancer mouse model with an intranasal administration of AD‐Cre induced KRAS G12D mutation, workflow diagram illustrating the single‐cell sequencing process, from sample collection to data analysis, and images of collected tissue samples, showcasing the distinct histopathological features identified through immunohistochemistry staining. (B, C) 2D uniform manifold approximation and projection visualization of all CD45^+^ (left) and CD45^−^ (right) cells across lung or tumour tissue, coloured according to main cell type (B) and subtypes (C). (D) A heatmap showing the expression levels of IFGRNS across various cell subtypes in the lung cancer mouse model, comparing samples across the different stages. Each row represents a cell subtype, with expression values normalized and scaled for clear comparison. Adjacent to the heatmap, a bar summarizes the aggregate IFGRNS expression for each cell subtype across all samples.

Utilizing a lung cancer mouse model with an intranasal administration of AD‐Cre‐induced KRAS G12D mutation, we assessed the alterations in IFGRNS scores during the tumour's growth phase, employing single‐cell sequencing to delineate the changes within the tumour and its microenvironment across distinct cell types. We strategically selected time points that align with critical stages in the development of lung adenocarcinoma (Figure 5A,B). At 2 weeks, we observed the onset of atypical adenomatous hyperplasia, which then progressed to the formation of small adenomas by 6 weeks. By 12 weeks, we noted significant tumour growth, and by 20 weeks, the mice had developed full adenocarcinoma. The single‐cell data analysis of those samples revealed a striking pattern of IFGRNS expression (Figure 5C–E; Figure S12). It was predominantly expressed in myeloid cells, with a particular abundance in M2 macrophages. Notably, the expression levels of IFGRNS in M2 macrophages and monocytes were found to be significantly elevated when compared with the normal lung tissue and the 2‐week samples (Figure 5E). This elevation in expression suggests a potential role of IFGRNS in the early stages of tumour development, possibly through the modulation of the tumour microenvironment by M2 macrophages. Further analysis of M2 macrophage subtypes revealed that IFGRNS was enriched in the M2b/d subtypes, as indicated by their cellular markers. These M2b/d macrophages were CD206‐negative and exhibited high expression of VEGF, Cd86, and TNF (Figure S13). Given that M2b/d macrophages are known to promote tumour progression and immune regulation, the correlation between the heightened expression of IFGRNS in these cells and the progression of LUAD provides a plausible link to the negative impact of IFGRNS on patient prognosis.

We sorted out the correlation between IFGRNS scores and tumour‐infiltrating immune cells in all patients. The results showed the IFGRNS score was significantly positively correlated with inflammation, TGF‐β‐associated ECM and macrophage and was significantly negatively correlated with T helper cells NK cells and CD8+ T cells in the tumour microenvironment (Table 2). These results suggest that tumour tissues from patients with higher IFNG scores exhibit immunosuppression. We found that IFGRNS exhibits a strong correlation with cancer‐associated fibroblast (CAF) activation and the activated stromal cell pathway in the TCGA cohorts (Table 2). Thus, we further discern the relationship between IFGRNS and the various CAF subtypes in single‐cell data from the 14 human breast cancer specimens. Our single‐cell analysis confirmed that IFGRNS is enriched in tumour‐associated macrophages (TAMs) and monocytes within breast cancer specimens (Figure S14A). In TAMs, the M2b/d subtypes exhibited high IFGRNS expression along with low CD206 levels and elevated expression of VEGF, CD86, and TNFSF14 (Figure S14B,C). Notably, IFGRNS scores were positively correlated with the proportion of interferon‐responsive CAFs within the tumour (*R* = .5, *p* = .12). In contrast, a negative correlation was observed with the proportion of dividing CAFs (*R* = −.54, *p* = .11; Figure S14D). Although the correlations did not reach statistical significance, likely due to the small sample size, they provide preliminary evidence of the association between IFGRNS and specific CAF subtypes.

**TABLE 2:** Correlation between the IFGRNS score and immunological features in all patients.^a^

Features	Correlation coefficient	*p*‐value	Adjusted *p*‐value
Inflammation	.4	0.00E+00	0.00E+00
TGF‐β‐associated ECM	.3	4.30E‐183	2.30E‐182
Activated dendritic cell	.28	2.60E‐166	1.30E‐165
Activated stromal cell	.26	9.30E‐144	3.50E‐143
Gamma delta T cell	.25	9.40E‐133	3.20E‐132
Neutrophil	.25	3.80E‐132	1.20E‐131
Macrophage	.25	6.10E‐126	1.70E‐125
Type 2 helper T cell	.21	2.20E‐87	4.30E‐87
Cancer‐associated fibroblast stimulated	.17	1.40E‐56	2.50E‐56
Type 1 helper T cell	.16	1.30E‐51	2.30E‐51
Regulatory T cell	.11	1.80E‐26	2.70E‐26
Dendritic cell	.09	5.40E‐17	6.80E‐17
Natural killer cell with high expression of CD56	.05	2.90E‐07	3.50E‐07
Natural killer cell with low expression of CD56	.03	2.80E‐03	3.20E‐03
Cytotoxic T cell	−.01	4.80E‐01	5.00E‐01
B cell	−.03	4.50E‐03	4.80E‐03
T cell	−.03	3.00E‐03	3.20E‐03
Normal stromal cell	−.04	2.60E‐05	3.00E‐05
Immature dendritic cell	−.08	1.20E‐14	1.50E‐14
Mast cell	−.1	2.40E‐20	3.10E‐20
Plasmacytoid dendritic cell	−.14	3.30E‐39	5.30E‐39
Central memory T cell	−.16	8.40E‐51	1.40E‐50
Effector memory T cell	−.17	3.50E‐57	6.60E‐57
CD8+ T cell	−.23	3.10E‐108	7.40E‐108
Natural killer cell	−.24	1.40E‐117	3.50E‐117
Eosinophil	−.25	3.90E‐126	1.10E‐125
Helper T cell	−.28	1.00E‐161	4.40E‐161
Type 17 helper T cell	−.32	1.50E‐218	9.30E‐218
Follicular helper T cell	−.39	0.00E+00	0.00E+00

^a^ The feature scores were evaluated by the single‐sample gene set enrichment analysis (ssGSEA) method for the specific gene sets. Correlations were tested using the Spearman correlation test. Adjusted *p*‐value is the *p*‐value after correction for multiple testing, using the false discovery rate (FDR) method.

### 3.6 IFGRNS scores are predictive of anti‐PD‐1 immunotherapy outcomes

**FIGURE 6:**
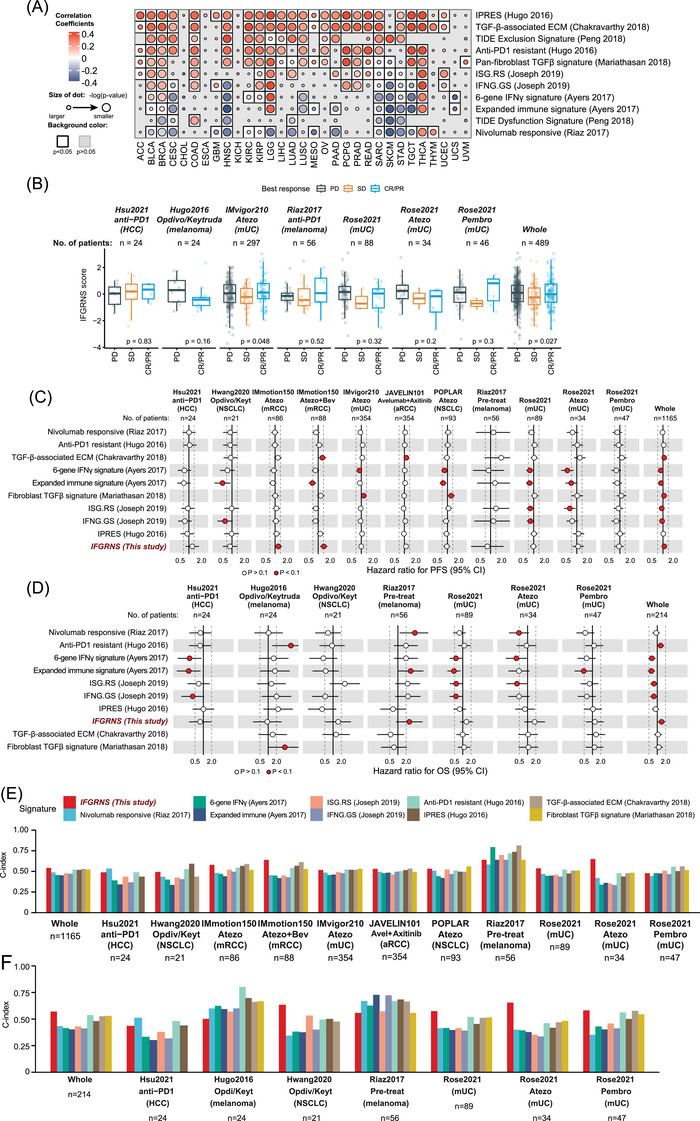
The correlation of IFGRNS score to the anti‐PD1 therapy resistance. (A) A heatmap showing the correlations of IFGRNS score to the Ayers IFN‐γ signature, Mariathasan pan‐fibroblast TGF‐β signature, Chakravarthy TGF‐β‐associated ECM, Hugo IPRES, Riaz nivolumab responsive signature, Joseph ISG resistance signature (ISG.RS), and Peng TIDE signature in the TCGA cohorts. The size of the dot indicates the p values. The filled colour of the dot indicates the correlation coefficient. The white colour of the background indicates the significance (*P* < .05) of the correlation. (B) Boxplots showing the differences in IFGRNS scores across tumour regression response in the clinical trial cohorts with the anti‐PD1 therapy. The upper, middle, and lower hinges of the box plot are 75th, 50th, and 25th quartiles, and the whiskers extend to the range below and above, respectively. CR, complete response; PR, partial response; SD, stable disease; PD, progressive disease. (C) Hazard ratio and 95% confidence interval (CI) for progression‐free survival of the signature scores in the cohorts with the anti‐PD1 therapy. (D) Hazard ratio and 95% confidence interval (CI) for overall survival of the signature scores in the cohorts with the anti‐PD1 therapy. (E, F) Comparison of C‐index for IFGRNS and other ICI‐Responsive signatures in predicting progression‐free survival (E) and overall survival (F) for patients treated with anti‐PD‐1 therapy. mUC, metastatic urothelial cancer; HCC, hepatocellular carcinoma; Atezo, atezolizumab; Pembro, pembrolizumab. Opdivo is also the nivolumab, and Keytruda is also the pembrolizumab.

Given that the IFGRNS scores correlated with tumour mutation burden and the tumour microenvironment, which are key factors that modulate the efficacy of ICI immunotherapy, particularly those targeting the PD‐1/PD‐L1 pathway—we speculated that IFGRNS may also serve as a predictive biomarker of response to ICI immunotherapy. Therefore, we compared the performance of the IFGRNS with several gene signatures associated with responses to ICI. Interestingly, we observed a positive correlation between IFGRNS scores and the signatures of resistance to anti‐PD‐1 immunotherapy (Hugo IPRES, Chakravarthy TGF‐β‐associated ECM, Peng TIDE signature, Joseph ISG.RS, and Mariathasan pan‐fibroblast TGF‐β signature) while a negative correlation was observed with responsiveness signatures (Ayers IFN‐γ signature, TIDE dysfunction, and Riaz nivolumab responsive signature) (Figure 6A) in several cancer types. These results suggested that IFGRNS may reflect the immunosuppressive mechanism of tumours, which may impair anti‐tumour immunity and limit the efficacy of ICI immunotherapy.

To further evaluate the impact of IFGRNS on the therapeutic response to anti‐PD‐1 immunotherapy, we analyzed IFGRNS scores in patients from clinical trial cohorts. We observed that higher IFGRNS scores were associated with poorer prognosis and lower tumour regression rates in urologic cancers, such as uroepithelial and renal cell carcinomas. In contrast, the correlation between IFGRNS and immunotherapy response was weaker in non‐small‐cell lung cancers, hepatocellular carcinoma, and melanomas (Figure 6B–D; Figure S15). We also compared IFGRNS with other ICI‐responsive signatures using metrics such as C‐index and AUC. IFGRNS consistently showed higher predictive power and robustness across several cancer types (e.g., melanoma, uroepithelial and renal cell carcinomas) (Figures S16–18; Figure 6C–F). Notably, it outperformed other ICI‐resistance markers in predicting survival in non‐ICI‐treated patients across cancer types (Figure S19).

**FIGURE 7:**
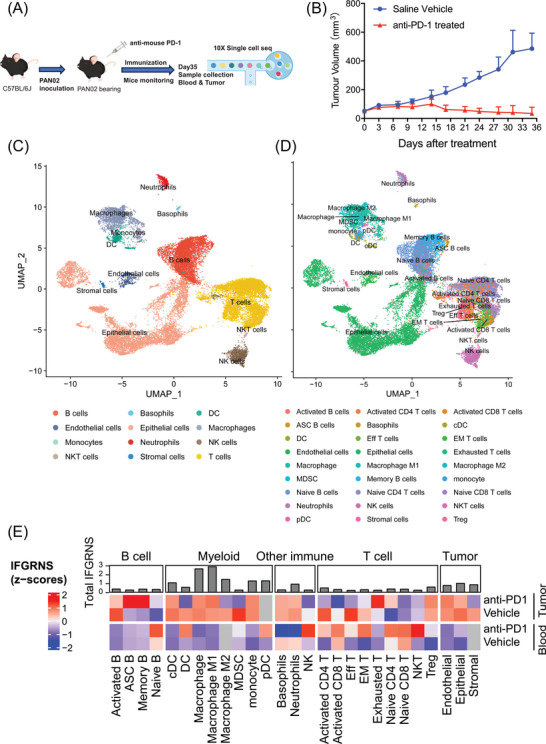
Single‐cell transcriptome sequencing of murine pancreatic cancer model treated with anti‐PD‐1. (A) The design of the pancreatic cancer mouse model treated with anti‐PD‐1, sample collection and single‐cell sequencing. (B) The effect of anti‐PD‐1 therapy on the growth of the PAN02 allografts in C57 mice. Data shown are mean + SD. (C, D) 2D uniform manifold approximation and projection visualization of all cells across both PBMCs and tumour tissue, coloured according to main cell type (C) and subtypes (D). (E) A heatmap showing the expression levels of IFGRNS across various cell subtypes in the pancreatic cancer mouse model, comparing samples treated with anti‐PD1 to untreated controls. Each column represents a cell subtype, with expression values normalized and scaled for clear comparison. Adjacent to the heatmap (top), a bar summarizes the aggregate IFGRNS expression for each cell subtype across all samples.

To validate the association between IFGRNS and outcomes of anti‐PD1 therapy. We designed an experiment utilizing subcutaneously implanted allografts derived from murine pancreatic cancer PAN02 cells in C57 mice. We collected blood and tumour samples from both treated and untreated groups and applied single‐cell transcriptome sequencing to capture the nuanced changes in IFGRNS expression (Figure 7A). Mice receiving anti‐PD1 treatment exhibited a significant reduction in tumour size as compared with the untreated group (Figure 7B). Further single‐cell analysis of IFGRNS distribution revealed a predominant enrichment within the macrophage population (Figure 7C–E; Figure S20). Among M2 macrophages, the subclusters that were CD206‐negative and expressed high levels of VEGF or TNF also showed greater IFGRNS expression (Figure S21). More compelling was the observation that upon anti‐PD1 therapy, the expression of IFGRN within M2 macrophages was markedly diminished (Figure 7E). Such findings confirm the association between the IFGRNS levels and therapeutic outcomes to anti‐PD1 treatment and suggest that anti‐PD1 therapy may exert its tumour‐suppressive effects, at least in part, by modulating IFGRNS expression in these cells.

### 3.7 IFGRNS signature enriched in genes involved in negative regulation of cytokine

**FIGURE 8:**
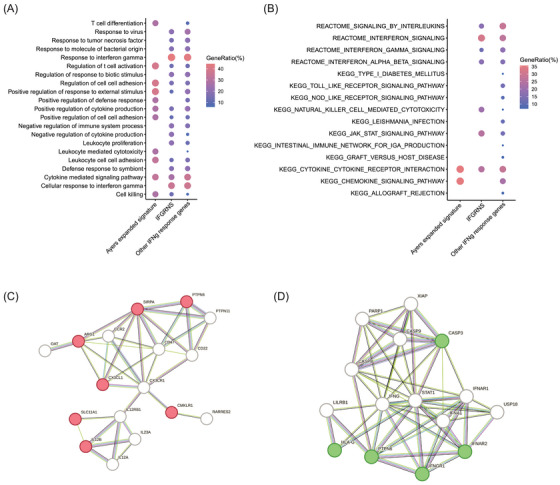
Functional and pathway analysis of IFN‐γ response genes in different subsets. (A) Gene ontology (GO) enrichment analysis of the genes involved in the IFGRNS, Ayers IFN‐γ signature, and other remaining IFN‐γ response genes. (B) Pathway (KEGG and REACTOME terms) enrichment of the same gene subsets. (C) The gene interaction network of the IFGRNS genes belonging to the GO term of negative regulation of cytokine production. (D) The gene interaction network of the IFGRNS genes belonging to the KEGG pathway of natural killer cell‐mediated cytotoxicity is displayed as a graph. The nodes represent genes and the edges represent interactions. Gene sets of GO and pathways were obtained from the GSEA database. Gene interaction relationships were obtained from the STRING database.

We explored the landscape of IFN‐γ regulated genes through two established signatures, ISG.RS and Ayers IFN‐γ, alongside our novel IFGRNS. Our IFGRNS signature emerged as distinct, sharing minimal overlap (three genes with ISG.RS and none with Ayers's). The ISG.RS has been posited as a resistance signature to anti‐PD‐1 therapy, predominantly expressed in cancer cells, yet influenced by IFNG.GS within CD8 T cells. Intriguingly, ISG.RS was positively correlated with its complementary subset IFNG.GS,^29^ suggesting a complex interplay rather than a straightforward antagonistic relationship. Moreover, ISG.RS, when assessed in bulk RNA sequencing data encompassing both tumour and microenvironment, did not necessarily confer resistance to survival or response to anti‐PD‐1 therapy (Figures S16–18). In contrast, IFGRNS was enriched in macrophages and stands as an independent prognostic indicator for poorer survival outcomes. Interestingly, the Ayers IFN‐γ signature had a positive correlation with better survival, which was contrary to the predictive power of the IFGRNS signature for prognosis. To investigate the underlying molecular mechanisms that account for this difference, we performed gene function and pathway enrichment analyses to compare the genes involved in the IFGRNS score with the Ayers IFN‐γ signature and the other IFN‐γ response genes. We found that the Ayers IFN‐γ signature genes were predominantly enriched in T cell activation and cell killing pathways, which are essential for anti‐tumour immunity. In contrast, the IFGRNS genes were specifically enriched in the function of negative regulation of cytokine (Figure 8A,B), which is a process that may dampen the immune response and confer tumour cells with survival and resistance advantages, leading to poor prognosis. We further constructed a regulatory network of those genes and identified four key regulators: CD47, CCR2, CD22, and CX3CR1 (Figure 8C,D). These genes are known to play important roles in inflammation, immunosuppression, and metastasis and tumour metastasis.^31^, ^32^, ^33^, ^34^

## 4 DISCUSSION

In this study, we systematically evaluated the pan‐cancer expression and prognostic implications of IFN‐γ response genes. IFN‐γ plays a dual role in tumour biology, promoting both anti‐tumour immunity and tumour progression. While IFN‐γ activates immune responses, such as M1 macrophage polarization, it can also contribute to immune evasion and resistance to immunotherapy under chronic stimulation. We identified 46 IFN‐γ response genes that are prognostic for survival across multiple cancer types and constructed an IFGRNS score based on their expression. The IFGRNS score was a robust prognostic biomarker for predicting DSS, OS, and PFI. However, its prognostic value varied across cancer types, being less predictive in pancreatic cancer (PAAD), MESO, and LGG. This variability may be attributed to differences in immune infiltration, tumour microenvironment composition, and genomic alterations. By integrating the IFGRNS score with clinical factors, such as AJCC staging, we developed a model that improved predictive accuracy across cancer types and demonstrated potential for clinical application.

Interestingly, IFGRNS represents a distinct signature compared with other IFN‐γ‐related gene sets, such as ISG.RS and the Ayers IFN‐γ signature. While the Ayers IFN‐γ signature is associated with better survival, IFGRNS is negatively correlated with outcomes, reflecting its enrichment in M2‐like macrophages, which are immunosuppressive and promote tumour progression.^35^, ^36^ These findings suggest that IFGRNS captures a pro‐tumorigenic aspect of IFN‐γ signalling in the tumour microenvironment, particularly in macrophage‐dominated tumours. We also found that anti‐PD1 therapy modulated the expression of IFGRNS, with reduced tumour size correlating with changes in IFGRNS levels, suggesting a potential mechanism of action for this therapy.

Further analysis showed that IFGRNS scores were negatively correlated with the infiltration of CD8+ T cells and NK cells in some cancers, indicating that IFGRNS is associated with an immunosuppressive environment that hinders immune cell infiltration. This was supported by single‐cell RNA sequencing, which revealed that IFGRNS is significantly expressed in M2 macrophages in lung adenocarcinoma and breast cancer. The M2 macrophage involved in this process is characterized by the M2b/d subtypes that were associated with immunosuppression and tumour promotion.

M2 macrophages are typically induced by Th2 cytokines such as IL‐4, IL‐13, and IL‐10, which are known to promote immunosuppressive and pro‐tumorigenic functions. Th2 cytokines, in contrast to IFN‐γ, induce an alternative activation pathway in macrophages, leading to the upregulation of markers such as CD206.^37^ Interestingly, while Th2 cytokines typically oppose IFN‐γ’s effects, our findings suggest a more complex interaction where M2 macrophages in the tumour microenvironment may exhibit hybrid phenotypes, co‐expressing both M2 markers (but CD206 negative/low) and IFN‐γ response genes. This hybrid phenotype may reflect a dynamic interplay within the tumour microenvironment, contributing to immune evasion and promoting tumour progression. This immunosuppressive environment can hinder the efficacy of immunotherapy; consistently, we observed a significant positive correlation between IFGRNS scores and the non‐response signature of anti‐PD1 treatment.

The IFN‐γ signalling pathway involves the binding of IFN‐γ to its receptor (IFNGR), the activation of Janus kinases (JAKs) and signal transducers and activators of transcription (STATs), especially STAT1, and the transcriptional regulation of IFN‐γ response genes.^18^, ^38^ However, the paradoxical role of IFN‐γ becomes evident under chronic signalling conditions, where persistent activation can lead to immune exhaustion and tumour resistance.^8^ This dual role is reflected in our study, where IFGRNS was found to be enriched in immunosuppressive myeloid cells, such as M2 macrophages, which have been associated with poor prognosis. IFN‐γ pathway also has extensive crosstalk with other cellular pathways, such as PI3K, MAPK/p38, and Toll‐like receptor signalling,^39^ which are involved in tumour progression and inflammation. The MHC molecules, which are induced by IFN‐γ and present antigens to T cells, initiate an adaptive immune response against tumour cells.^40^ However, tumour cells may evade this response by downregulating MHC expression or by expressing immunosuppressive molecules, such as PD‐L1.^41^, ^42^, ^43^ The ID) enzyme, which is induced by IFN‐γ and catalyzes the degradation of tryptophan, an essential amino acid for T‐cell proliferation and survival.^44^, ^45^ IDO may create an immunosuppressive microenvironment by depleting tryptophan, generating kynurenine metabolites, and inducing regulatory T cells (Tregs).^46^, ^47^ The immune checkpoint molecules, such as PD‐L1, CTLA‐4, LAG‐3, and TIGIT, are induced or regulated by IFN‐γ and modulate the activation or inhibition of T cells.^39^ These molecules may limit anti‐tumour immunity by inducing T‐cell exhaustion or anergy or by enhancing Treg function.^48^, ^49^ Those might be the possible mechanisms and pathways that mediate the effects of IFN‐γ response genes on tumour immunity and immunotherapy.

There are several limitations to the current study. One major challenge is the heterogeneity across public datasets like TCGA and other sources, which may affect the robustness and consistency of the IFGRNS model. Differences in sample collection, processing, and annotation across these datasets can introduce variability and noise, potentially impacting the reproducibility and comparability of our findings. Moreover, small sample sizes and event rates for certain cancer types in these datasets may limit the generalizability of the model. We explored the prognostic value of IFGRNS across external datasets. However, we observed some inconsistencies, for instance, in colorectal cancer cohorts. Our subgroup analyses revealed that variations in immune cell infiltration and TME composition may explain the differing prognostic significance of IFGRNS across different datasets. These insights reinforce the idea that the prognostic value of IFGRNS is heavily influenced by the gene expression and biological context of the tumours in certain datasets, suggesting that its application might need to be tailored to specific cancer types or combined with other markers for a more comprehensive predictive model. To mitigate these challenges, future studies should focus on validating the IFGRNS model in larger, more standardized datasets and explore its integration with clinical factors for prospective clinical applications.

In conclusion, our study provides a comprehensive analysis of IFN‐γ response genes across multiple cancer types, identifying 46 genes that are prognostic for disease‐specific survival. The IFGRNS score emerged as a robust predictor of survival outcomes and immunotherapy response, particularly reflecting the activity of myeloid cells, especially the M2 macrophage subtype, which promotes tumour progression. However, the prognostic value of IFGRNS varies across cancer types and tumour microenvironments. Future research should explore these mechanisms further to improve the score's clinical utility for personalized cancer therapy.

## AUTHOR CONTRIBUTIONS

Yuchao Zhang, Xiangyin Kong, and Landian Hu conceived and designed the study. Yuchao Zhang, Zhenchuan Wu, Hailong Wang, Hongyu Zhang, and Xiaoxue Li collected and assembled the data. Yuchao Zhang, Asma Khanniche, Yizhe Li, and Landian Hu analyzed and interpreted the data. All authors participated in writing the paper and approved the final version of the paper.

## CONFLICT OF INTEREST STATEMENT

All authors declare no conflict of interest.

## CONSENT TO PARTICIPATE

Not applicable.

## CONSENT FOR PUBLICATION

Not applicable.

### ETHICS STATEMENT

All animal experiments were approved by the Experimental Animal Management and Use Committee (IACUC) of the Shanghai Institute of Materia Medica, Chinese Academy of Sciences, and were conducted in accordance with the regulations set by the International Commission for the Evaluation and Accreditation of Experimental Animals (AAALAC).

## Supporting information



Supporting Information

## Data Availability

The availability of data and material were all described in the Materials and Methods section.
