# Supplementary material for: A myeloid IFN gamma response gene signature correlates with cancer prognosis
Source: Clin Transl Med. 2025 Mar 31;15(4):e70139. doi: 10.1002/ctm2.70139 (PMC11959096; doi:10.1002/ctm2.70139)

**A myeloid IFN gamma response gene signature correlates with cancer prognosis**

Yuchao Zhang ✉^1,*^, Asma Khanniche ^2,*^, Yizhe Li ^1,*^, Zhenchuan Wu ^1, 2^, Hailong Wang ^1, 2^, Hongyu Zhang ^1^, Xiaoxue Li ^1^, Landian Hu✉^1,2,3^, Xiangyin Kong✉^1,4^

^1^ CAS Key Laboratory of Tissue Microenvironment and Tumor, Shanghai Institute of Nutrition and Health, Chinese Academy of Sciences, Shanghai, China.

^2^ ANDA Biology Medicine Development (Shenzhen) Co., LTD, China.

^3^ Shenzhen Institutes of Advanced Technology, Chinese Academy of Sciences, Shenzhen, China.

^4^ School of Life Science and Technology, ShanghaiTech University, Shanghai, China.

*These authors contributed equally.

✉Coresponding authors: Xiangyin Kong ([xykong@sibs.ac.cn](mailto:xykong@sibs.ac.cn)), Landian Hu ([ldhu2013@163.com](mailto:ldhu2013@163.com)), and Yuchao Zhang ([zhangyuchao@sibs.ac.cn](mailto:zhangyuchao@sibs.ac.cn)).

**Table of content**

[Supplementary table 2](#_Toc179974358)

[Table S1 2](#_Toc179974359)

[Supplementary figures 3](#_Toc179974360)

[Figure S1 3](#_Toc179974361)

[Figure S2 4](#_Toc179974362)

[Figure S3 5](#_Toc179974363)

[Figure S4 6](#_Toc179974364)

[Figure S5 7](#_Toc179974365)

[Figure S6 8](#_Toc179974366)

[Figure S7 9](#_Toc179974367)

[Figure S8 10](#_Toc179974368)

[Figure S9 11](#_Toc179974369)

[Figure S10 12](#_Toc179974370)

[Figure S11 13](#_Toc179974371)

[Figure S12 14](#_Toc179974372)

[Figure S13 15](#_Toc179974373)

[Figure S14 16](#_Toc179974374)

[Figure S15 17](#_Toc179974375)

[Figure S16 18](#_Toc179974376)

[Figure S17 19](#_Toc179974377)

[Figure S18 20](#_Toc179974378)

[Figure S19 21](#_Toc179974379)

[Figure S20 22](#_Toc179974380)

[Figure S21 23](#_Toc179974381)

## Supplementary table

### Table S1

**TCGA cancer types and their abbreviations.**

| **Abbreviation** | **Cancer types** |
| --- | --- |
| ACC | Adrenocortical Cancer |
| BLCA | Bladder Cancer |
| BRCA | Breast Cancer |
| CESC | Cervical Cancer |
| CHOL | Bile Duct Cancer |
| COAD | Colon Cancer |
| DLBC | Large B-cell Lymphoma |
| ESCA | Esophageal Cancer |
| GBM | Glioblastoma |
| HNSC | Head and Neck Cancer |
| KICH | Kidney Chromophobe |
| KIRC | Kidney Clear Cell Carcinoma |
| KIRP | Kidney Papillary Cell Carcinoma |
| LGG | Lower Grade Glioma |
| LIHC | Liver Cancer |
| LUAD | Lung Adenocarcinoma |
| LUSC | Lung Squamous Cell Carcinoma |
| MESO | Mesothelioma |
| OV | Ovarian Cancer |
| PAAD | Pancreatic Cancer |
| PCPG | Pheochromocytoma & Paraganglioma |
| PRAD | Prostate Cancer |
| READ | Rectal Cancer |
| SARC | Sarcoma |
| SKCM | Melanoma |
| STAD | Stomach Cancer |
| TGCT | Testicular Cancer |
| THCA | Thyroid Cancer |
| THYM | Thymoma (THYM) |
| UCEC | Endometrioid Cancer |
| UCS | Uterine Carcinosarcoma |
| UVM | Ocular melanomas |

## Supplementary figures

### Figure S1

**Flow chart of the IFGRNS score model construction and analysis.**


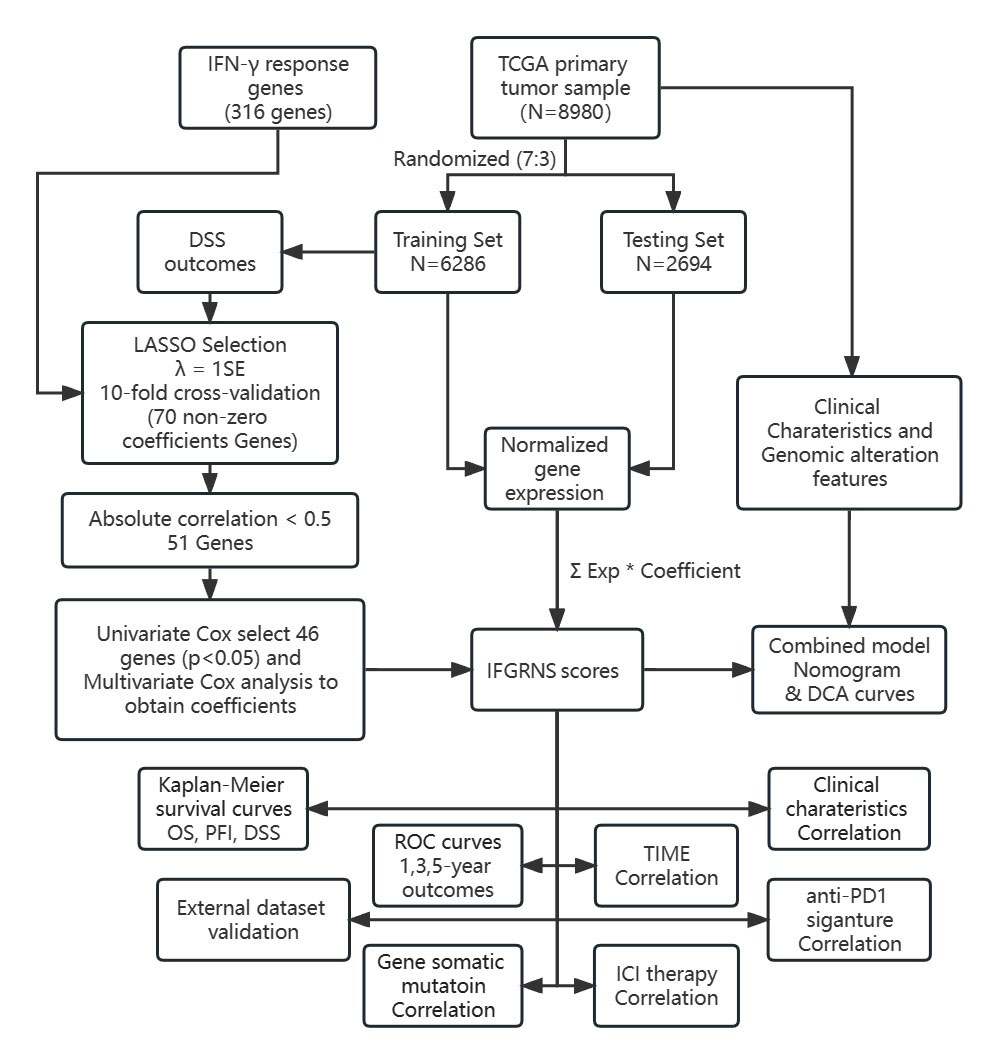


### Figure S2

**Correlation of gene expression between pairwise of the 70 genes derived from selection of the LASSO Cox regression analysis.** Dark background indicates the p value < 0.05. Nineteen genes (in red color) that were excluded to reduce pairwise correlations. The `findCorrelation` function in the caret package for R was used to search the correlation matrix and returns the genes that need to be removed. The pairwise absolute correlation threshold was chosen to be 0.5.


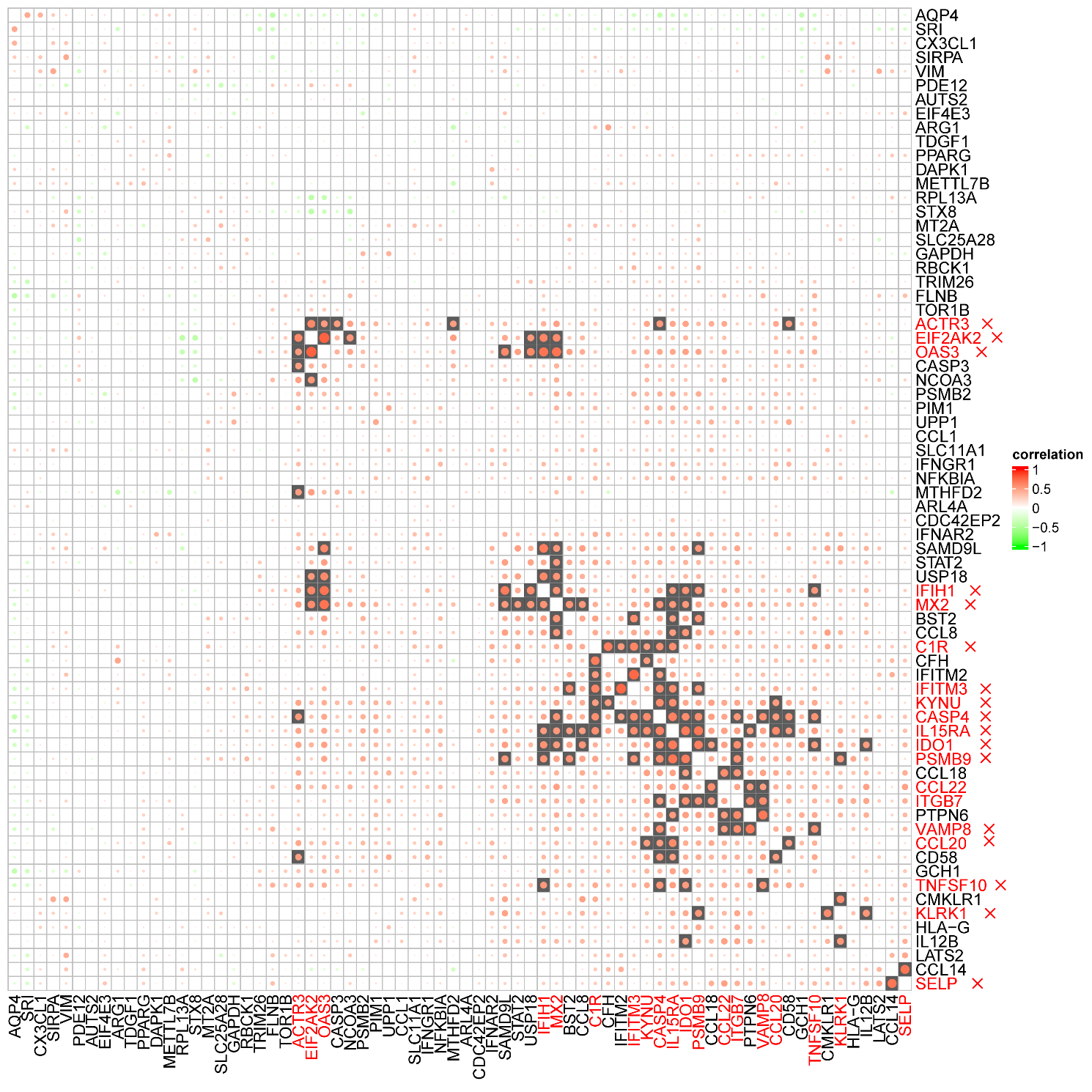


### Figure S3

**Kaplan-Meier survival curves for the disease specific survival of patients with high IFGRNS scores and low IFGRNS scores in each cancer type in training set.** The yellow curve indicates the high-score group and the blue curve indicates the low-score group. The High-score and low-score groups were classified by the median IFGRNS score. The colored area indicates the 95% confidence intervals.


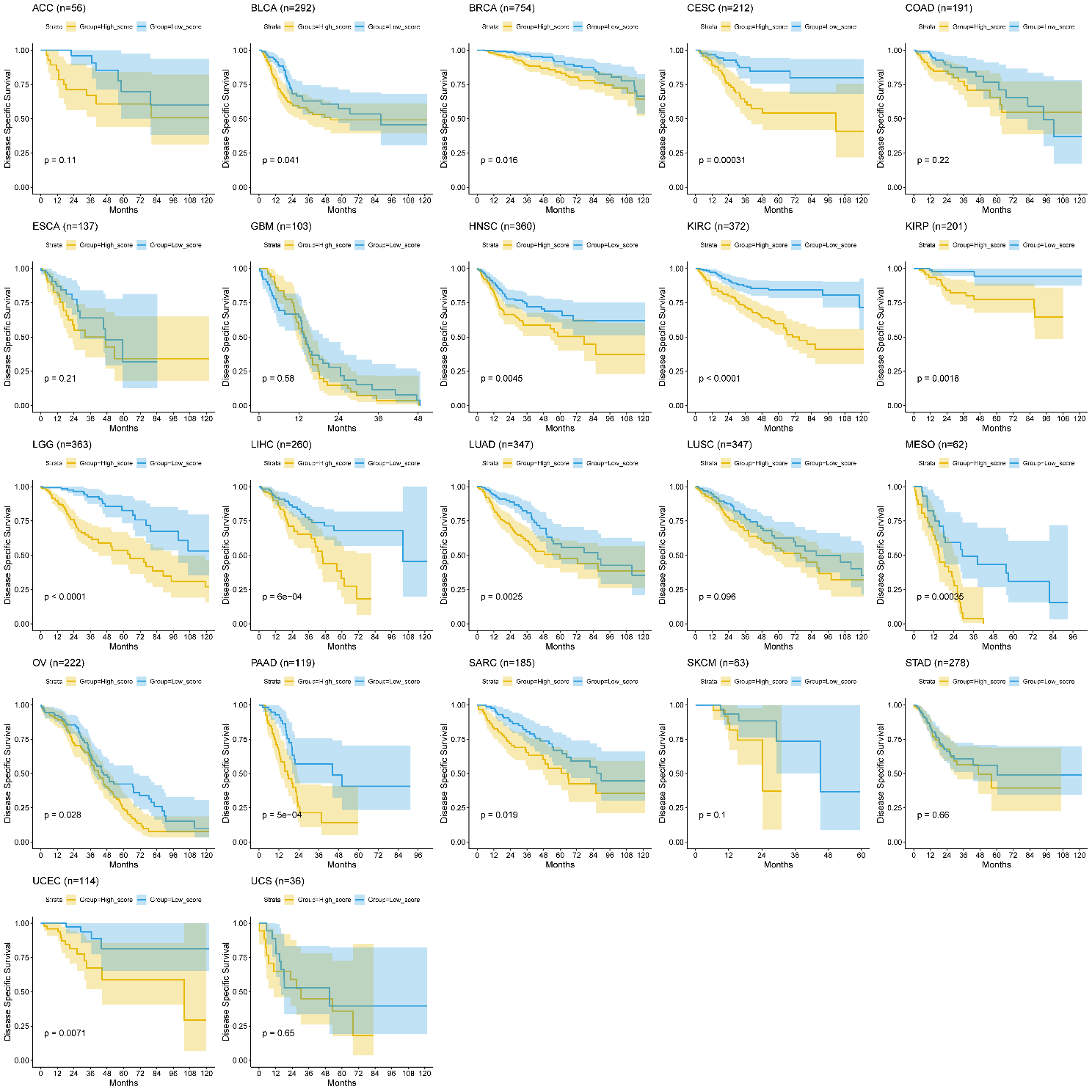


### Figure S4

**Kaplan-Meier survival curves for the overall survival of patients with high IFGRNS scores and low IFGRNS scores in each cancer type in training set.** The yellow curve indicates the high-score group and the blue curve indicates the low-score group. The High-score and low-score groups were classified by the median IFGRNS score. The colored area indicates the 95% confidence intervals.


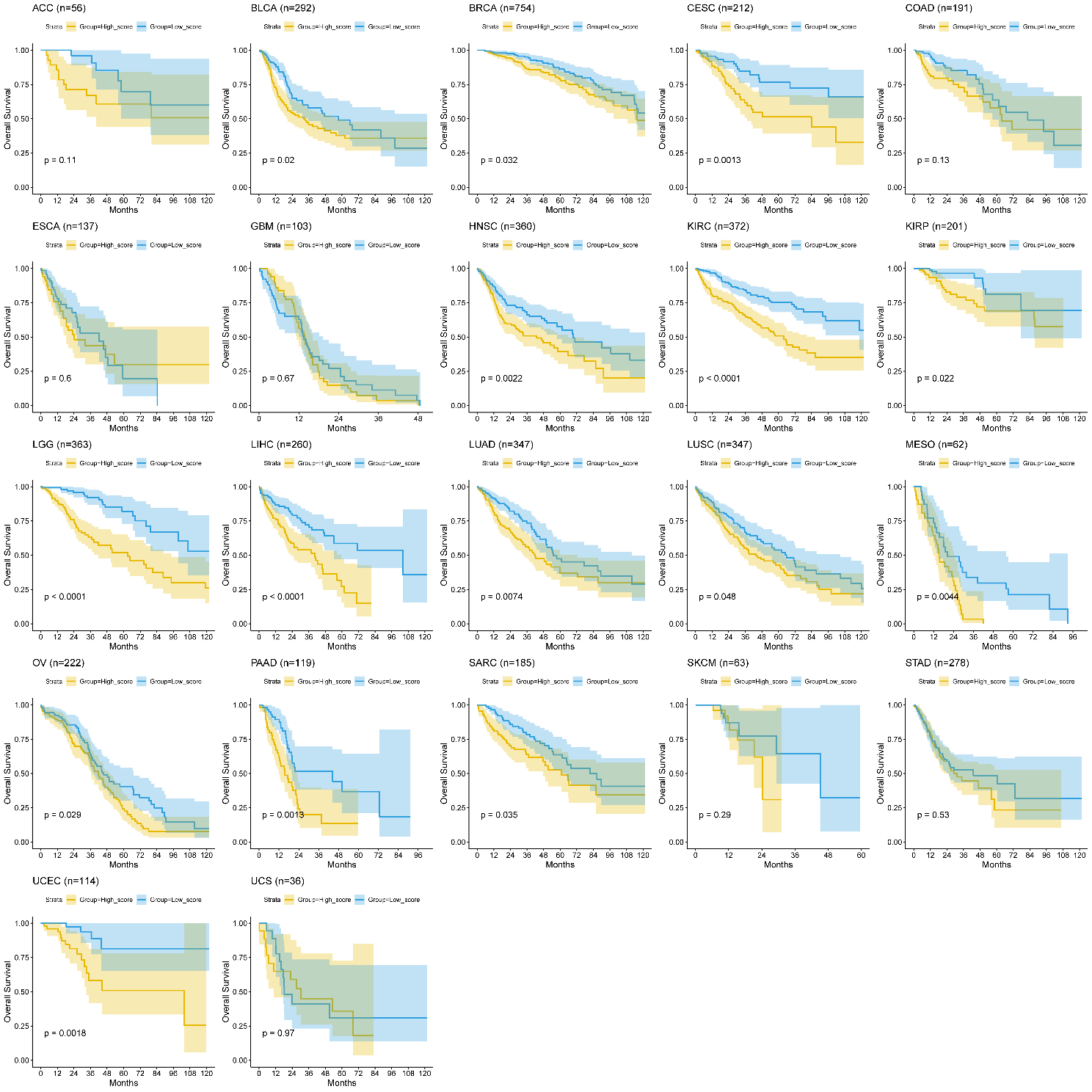


### Figure S5

**Kaplan-Meier survival curves for the progression free survival of patients with high IFGRNS scores and low IFGRNS scores in each cancer type in training set.** The yellow curve indicates the high-score group and the blue curve indicates the low-score group. The High-score and low-score groups were classified by the median IFGRNS score. The colored area indicates the 95% confidence intervals.


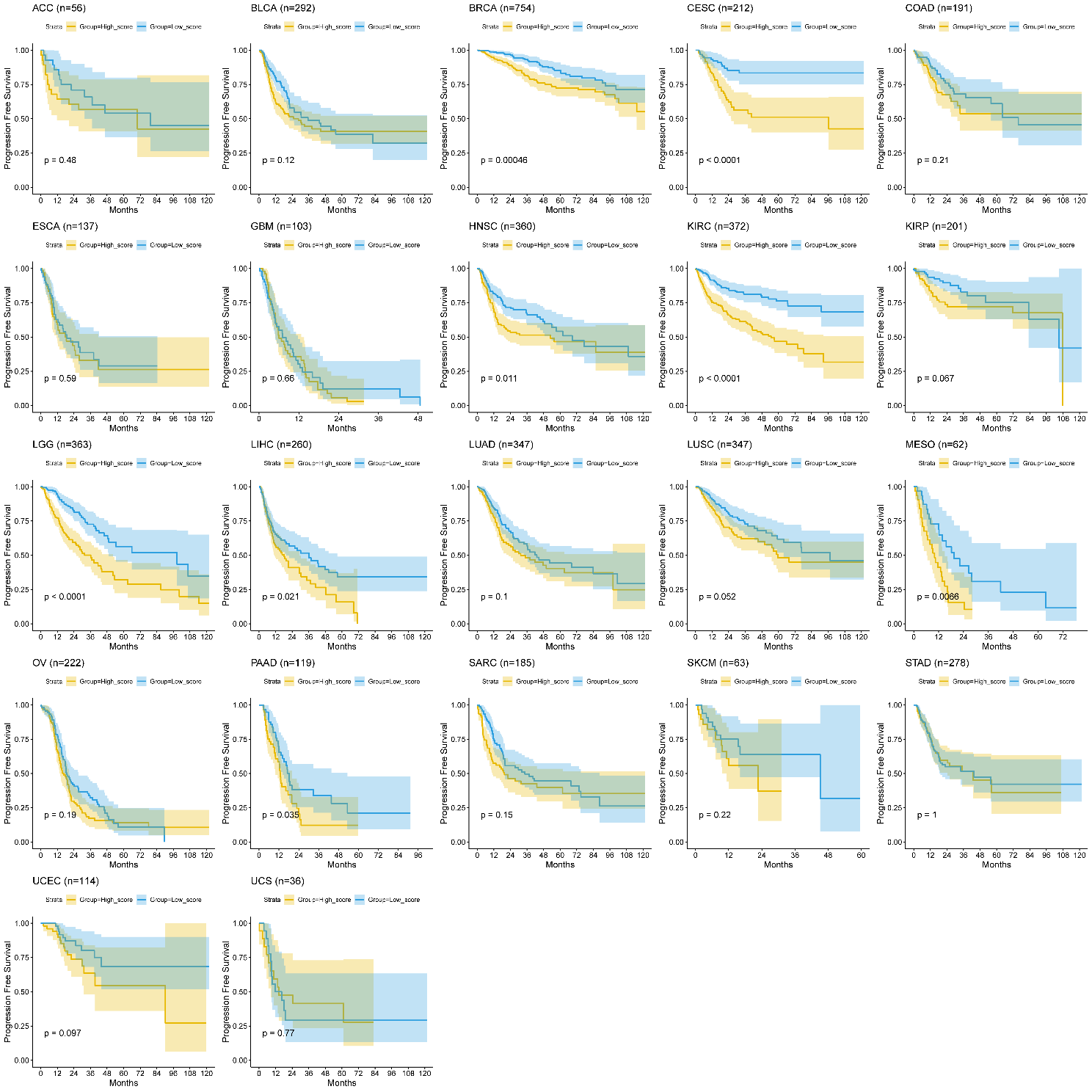


### Figure S6

**Kaplan-Meier survival curves for the disease specific survival of patients with high IFGRNS scores and low IFGRNS scores in each cancer type in testing set.** The yellow curve indicates the high-score group and the blue curve indicates the low-score group. The High-score and low-score groups were classified by the median IFGRNS score. The colored area indicates the 95% confidence intervals.


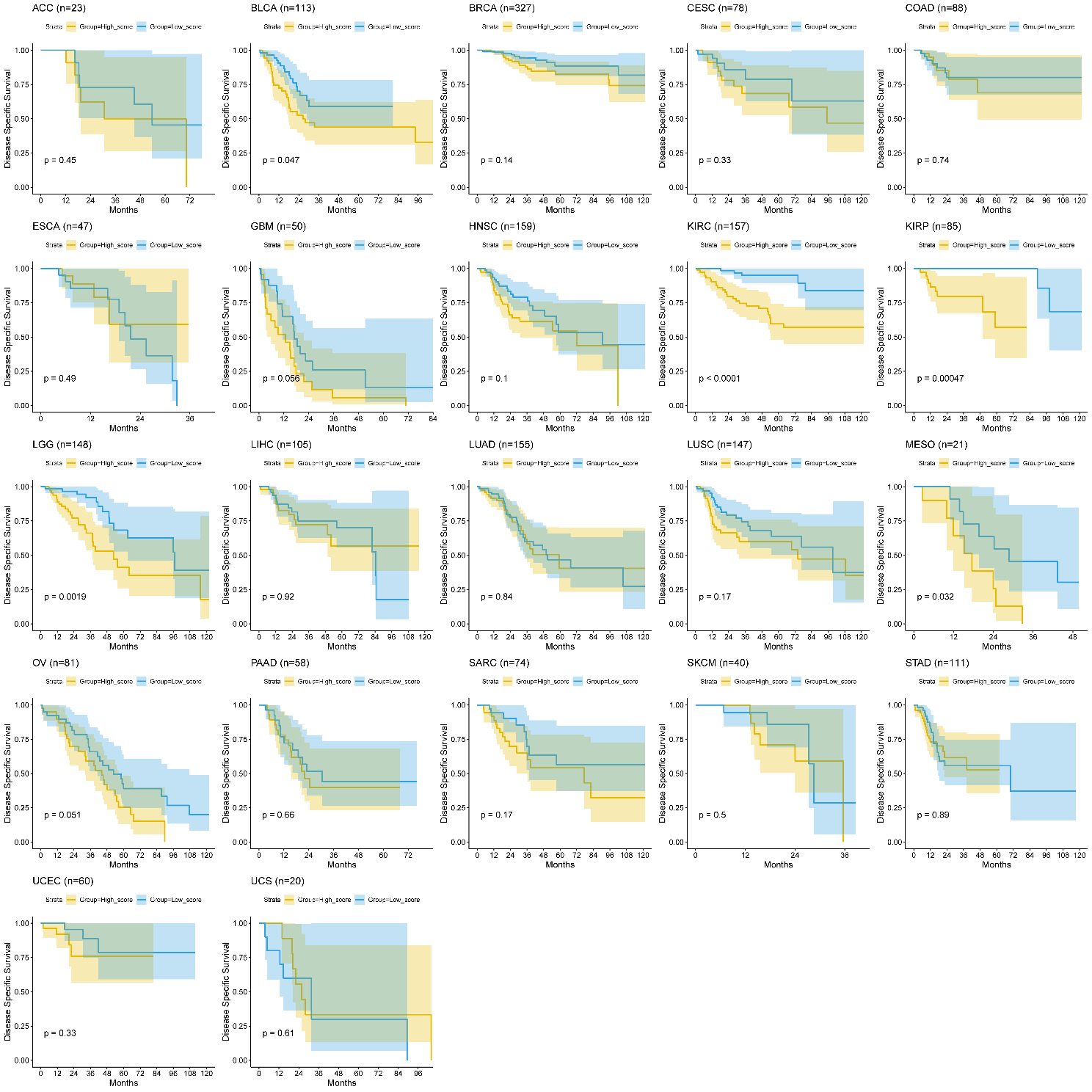


### Figure S7

**Kaplan-Meier survival curves for the overall survival of patients with high IFGRNS scores and low IFGRNS scores in each cancer type in testing set.** The yellow curve indicates the high-score group and the blue curve indicates the low-score group. The High-score and low-score groups were classified by the median IFGRNS score. The colored area indicates the 95% confidence intervals.


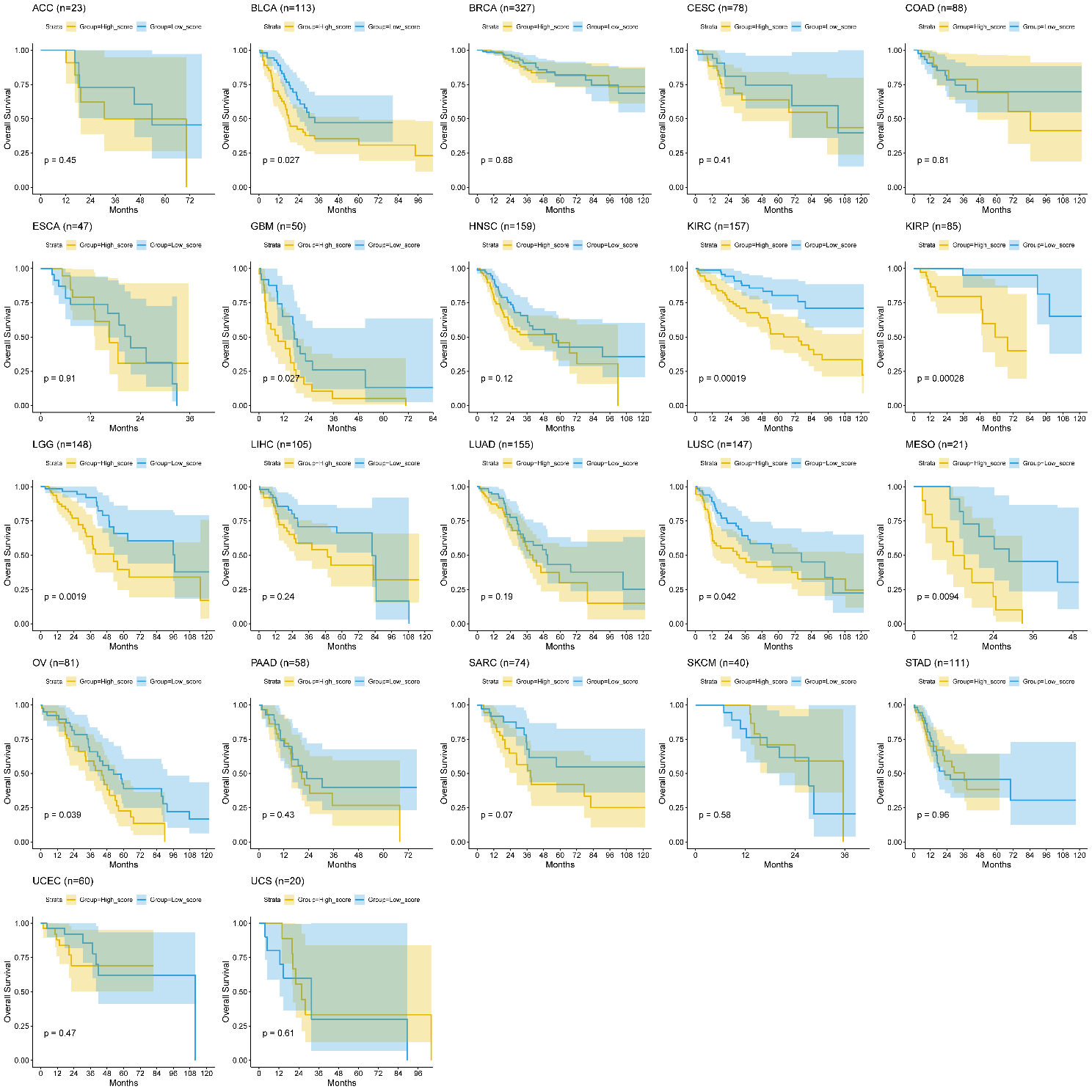


### Figure S8

**Kaplan-Meier survival curves for the progression free survival of patients with high IFGRNS scores and low IFGRNS scores in each cancer type in testing set.** The yellow curve indicates the high-score group and the blue curve indicates the low-score group. The High-score and low-score groups were classified by the median IFGRNS score. The colored area indicates the 95% confidence intervals.


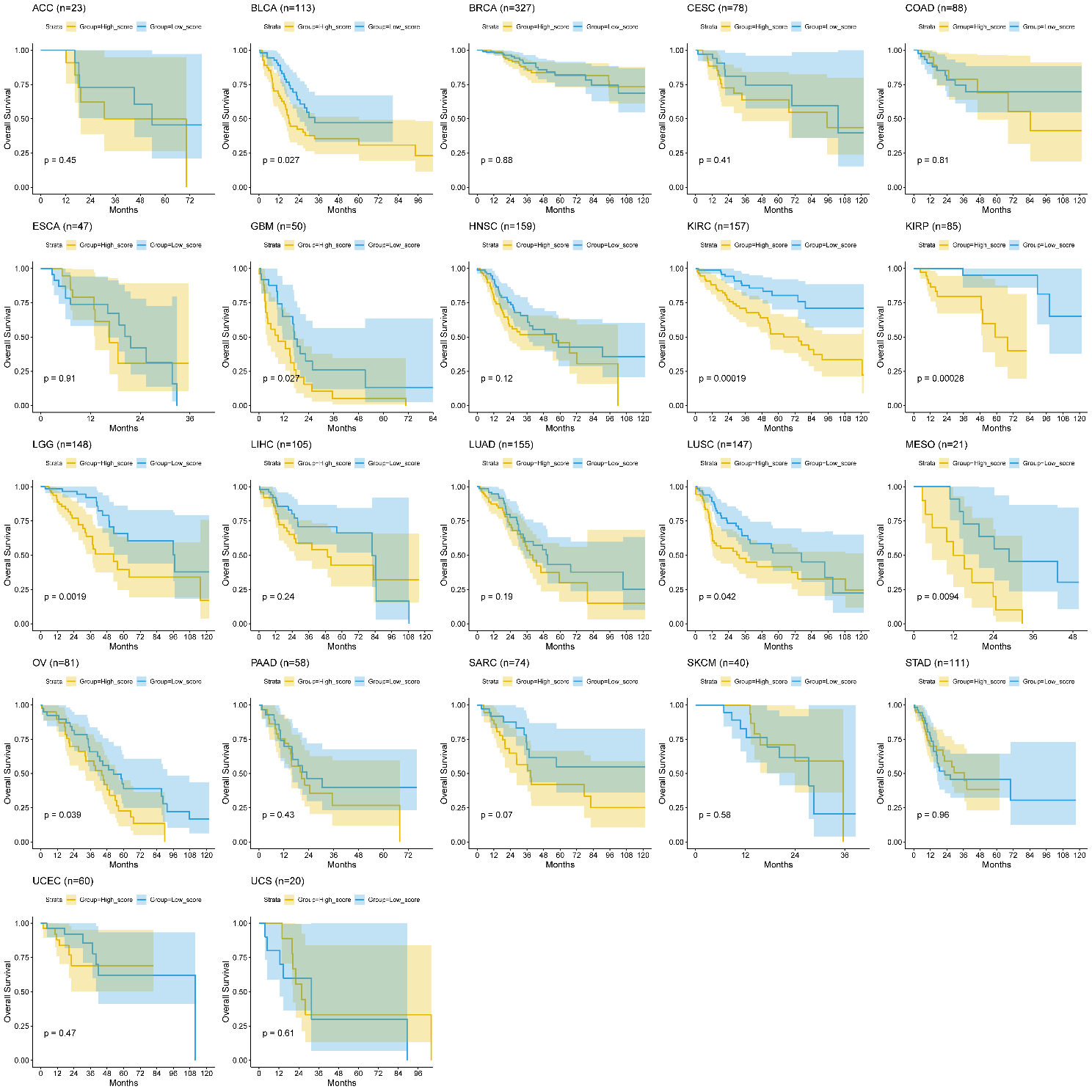


### Figure S9

**Differences in tumor microenvironment (TME) composition and gene expression between patients with high IFGRNS scores and low IFGRNS scores across the datasets of colorectal cancers. A**, TME composition and immune infiltration across cohorts. **B**, the boxplots showing the significant differences in the immune cells. The upper, middle, and lower hinges of the box plot are 75th, 50th, and 25th quartiles, and the whiskers extend to the range below and above, respectively. **C**, the gene oncology enrichment of significantly different expressed genes between two groups. **D-E**, gene set enrichment in cytokine-mediated signaling and cell chemotaxis.


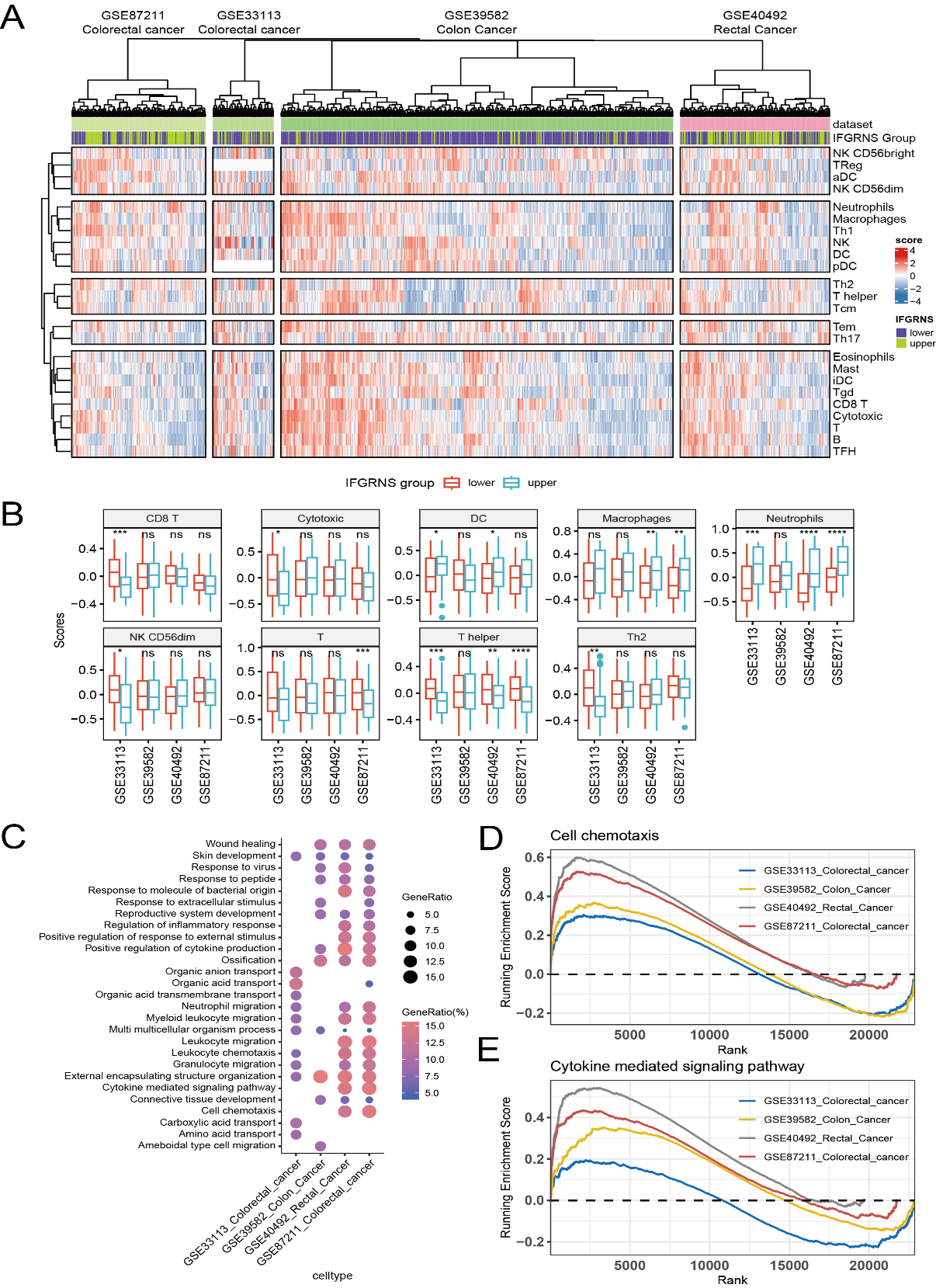


### Figure S10

**The correlations between the IFGRNS score and cancer types or subtypes in TCGA cohorts.**

**A**, the boxplots showing the significant differences in the IFGRNS score across the tumor pathology stage in BLCA, COAD, HNSC, KIRC, KIRP, LUAD, TGCT, and THCA. **B**, the boxplots showing the significant differences in the IFGRNS score across the tumor subtypes in BRCA, CESC, GBM, HNSC, LGG, SARC, STAD, TGCT, and UCEC. The upper, middle, and lower hinges of the box plot are 75th, 50th, and 25th quartiles, and the whiskers extend to the range below and above, respectively.


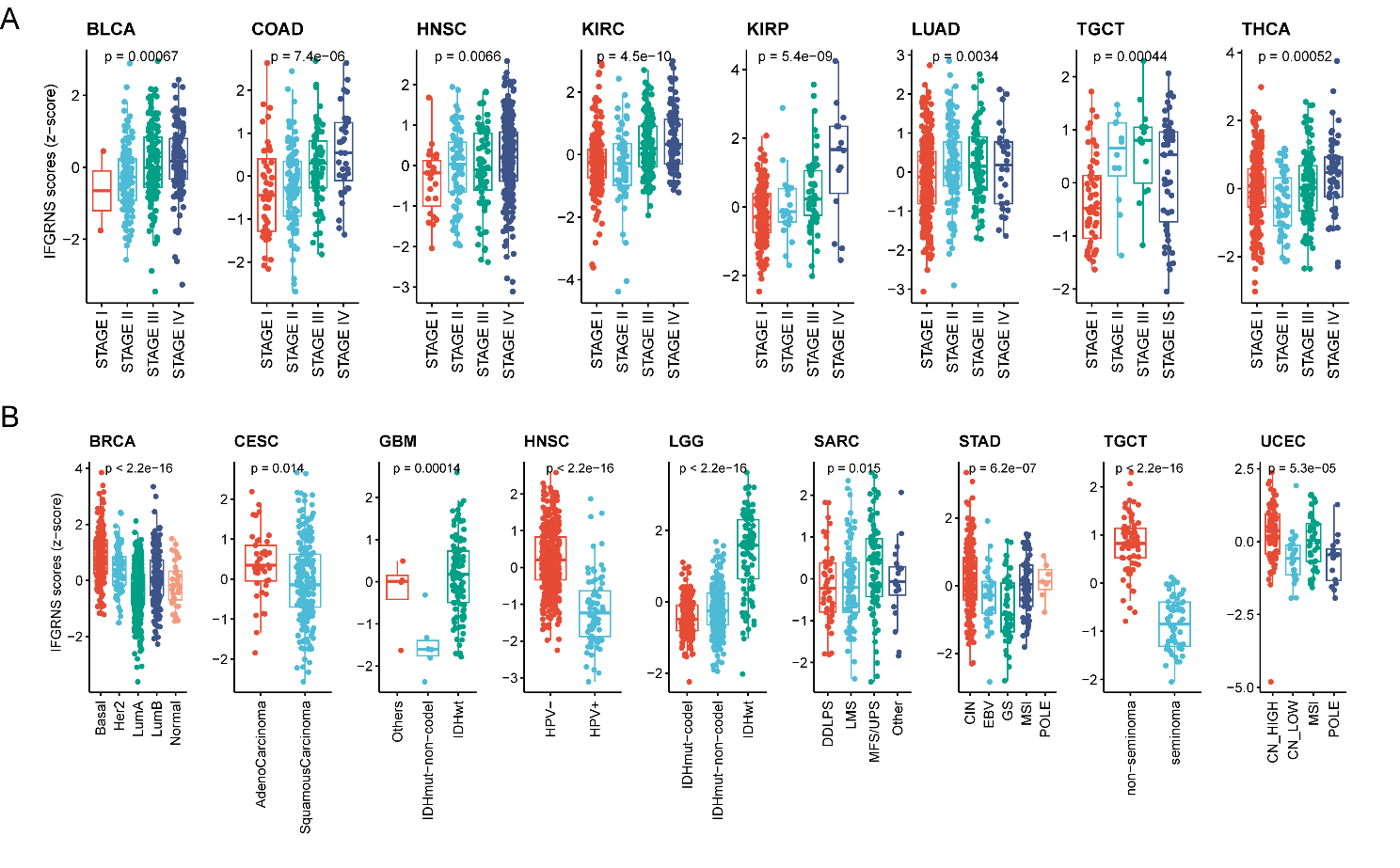


### Figure S11

**An overview of the association between clinical characteristics and the IFGRNS score in BLCA, HNSC, KIRC, KIRP, and THCA.** Columns represent samples sorted by IFGRNS score from low to high (top row). Rows represent clinical characteristics significantly associated with the IFGRNS score (p < 0.05).


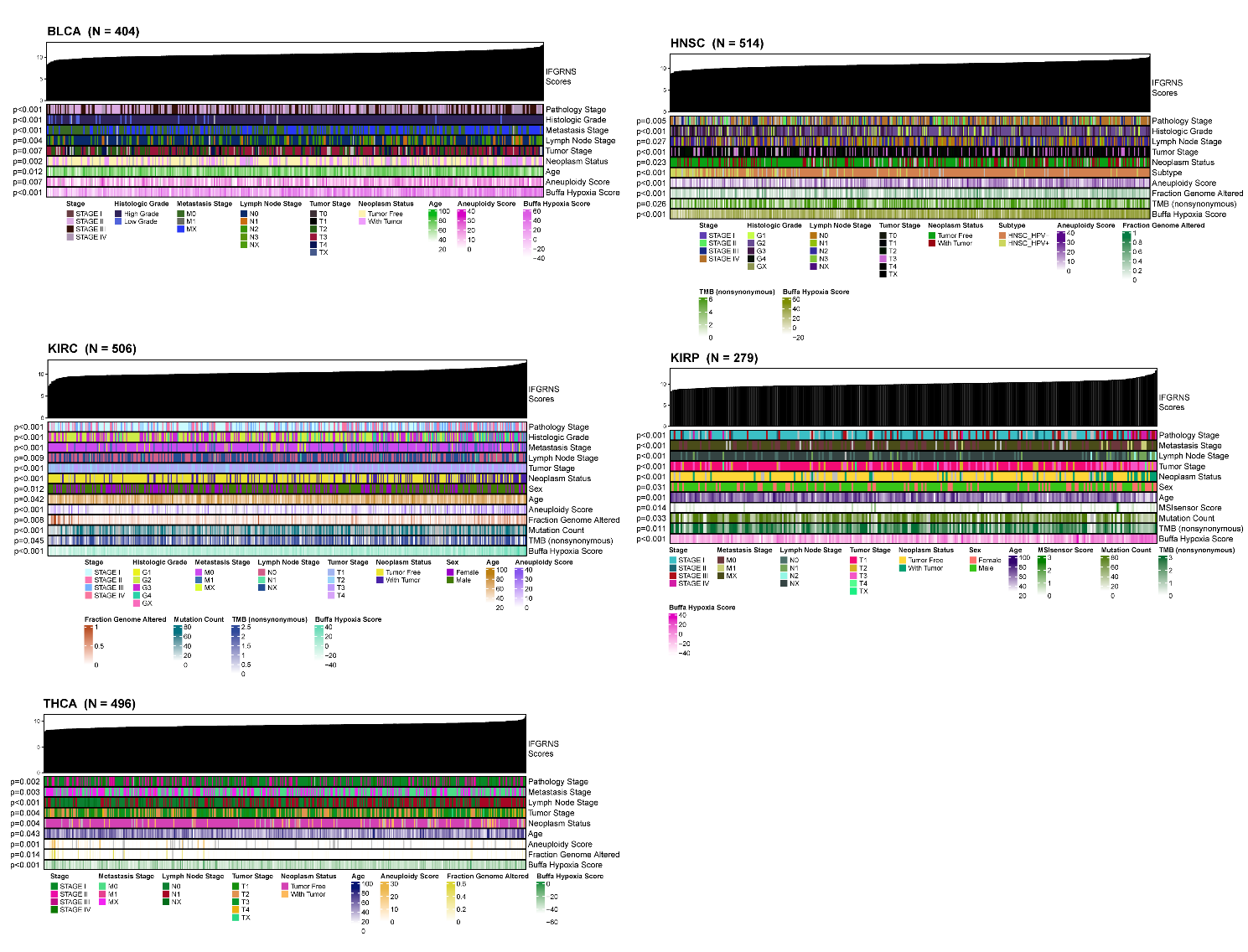


### Figure S12

**The single-cell data analysis of a lung cancer mouse model with an intranasal administration of AD-Cre induced KRAS G12D mutation. The heatmap indicating the subtypes of B cells (A), T cells (B), monocytes/DCs (C), and macrophages (D) and their marker genes for the classification.**


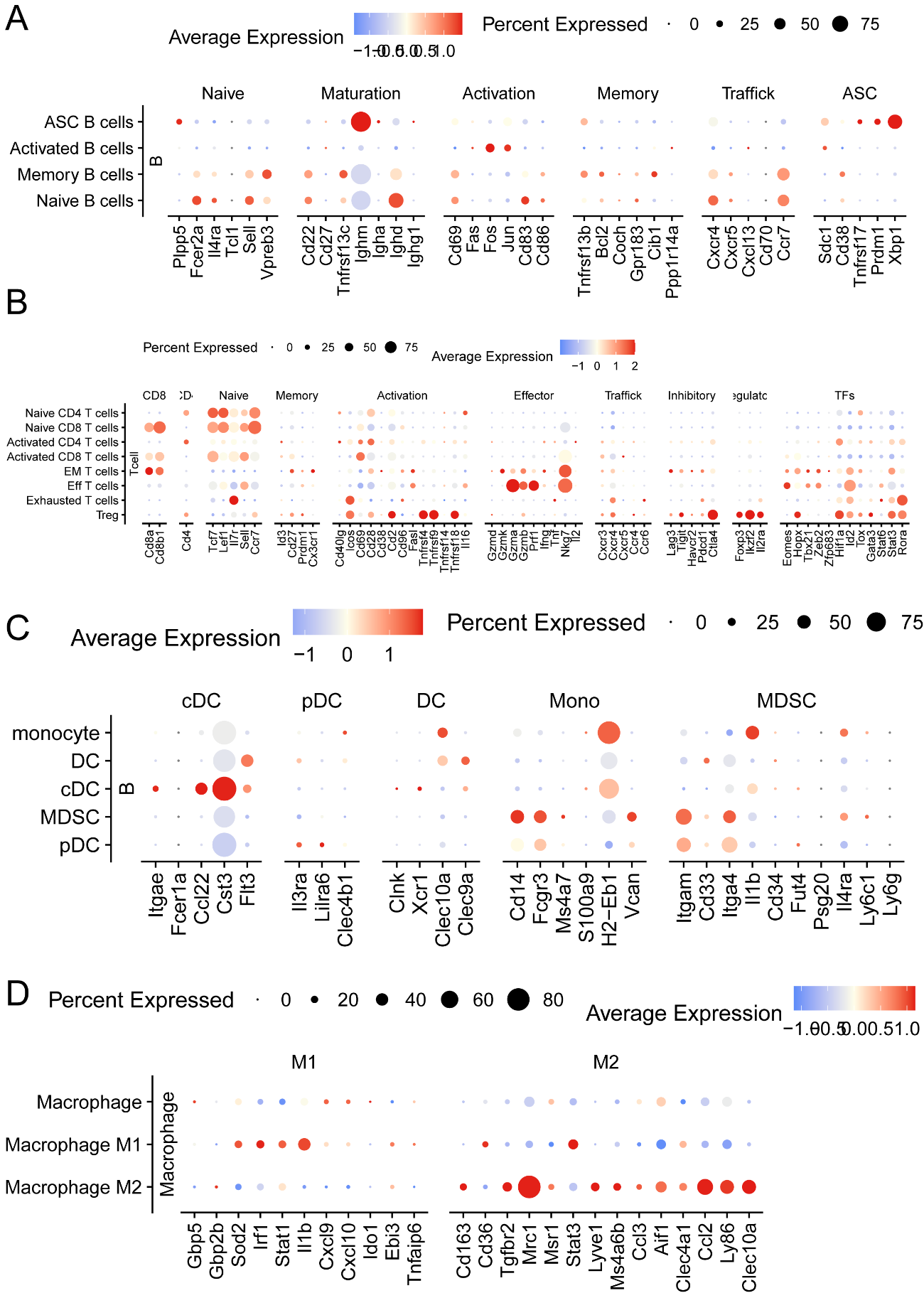


### Figure S13

**IFGRNS scores across the subclusters of the M2 macrophage in the lung cancer mouse model with an intranasal administration of AD-Cre induced KRAS G12D mutation. A,** 2D UMAP visualization of all M2 macrophages and subclusters. **B**, the dot heatmap indicating the subclusters and their marker genes for the classification. **C**, a heatmap showing the expression levels of IFGRNS across various subclusters, comparing samples across the different stages. Each row represents a subcluster, with expression values normalized and scaled for clear comparison. Adjacent to the heatmap, a bar summarizes the aggregate IFGRNS expression for each subcluster across all samples.


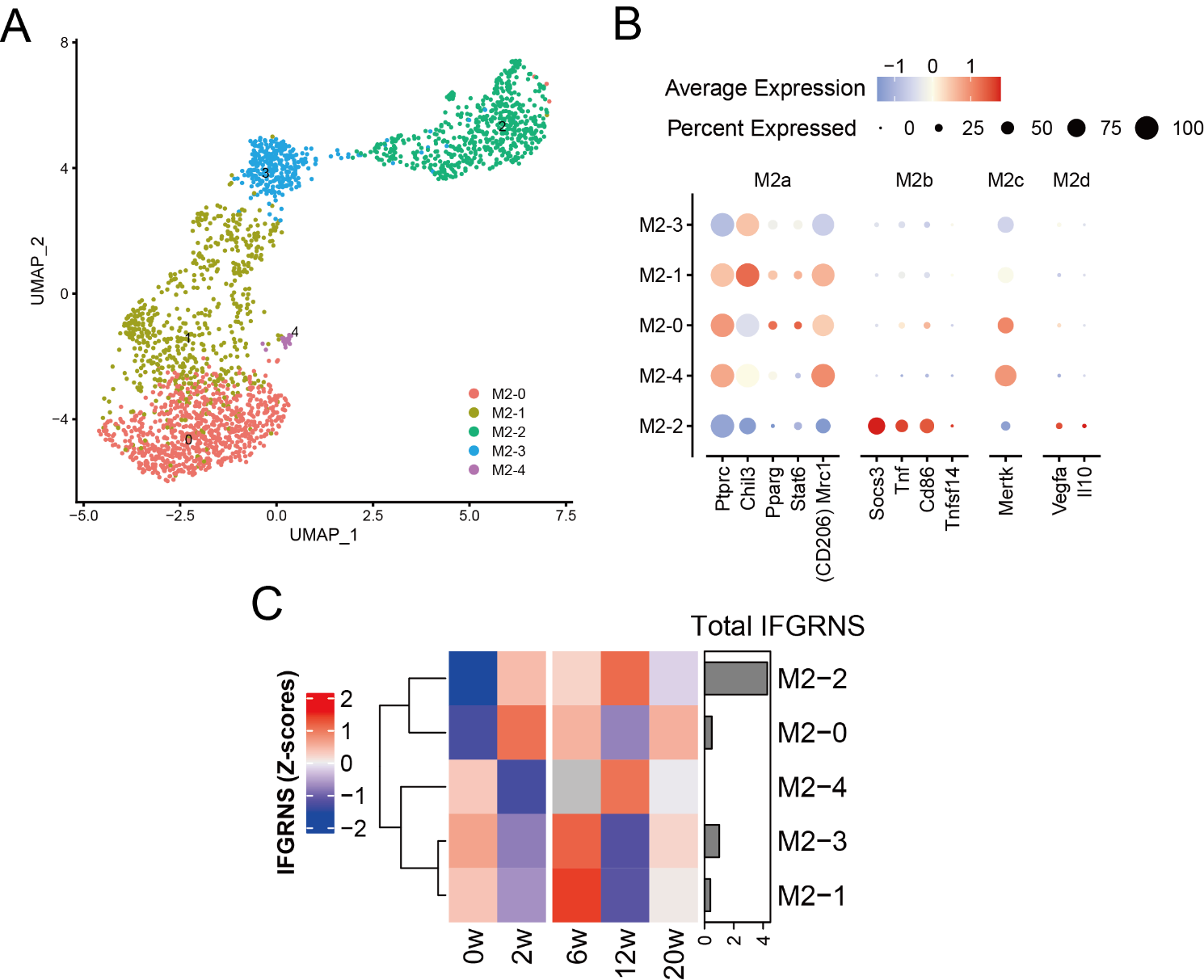


### Figure S14

**The correlation between IFGRNS and the various CAF subtypes in a single-cell data from the 14 human breast cancer specimens. A,** IFGRNS scores across each subtype of cells in tumors**. B,** the dot heatmap indicating the subclusters and their marker genes for the classification. **C**, a bar that summarizes the IFGRNS expression for each TAM subcluster. **D**, correlation between IFGRNS and the various CAF subtypes in tumors.


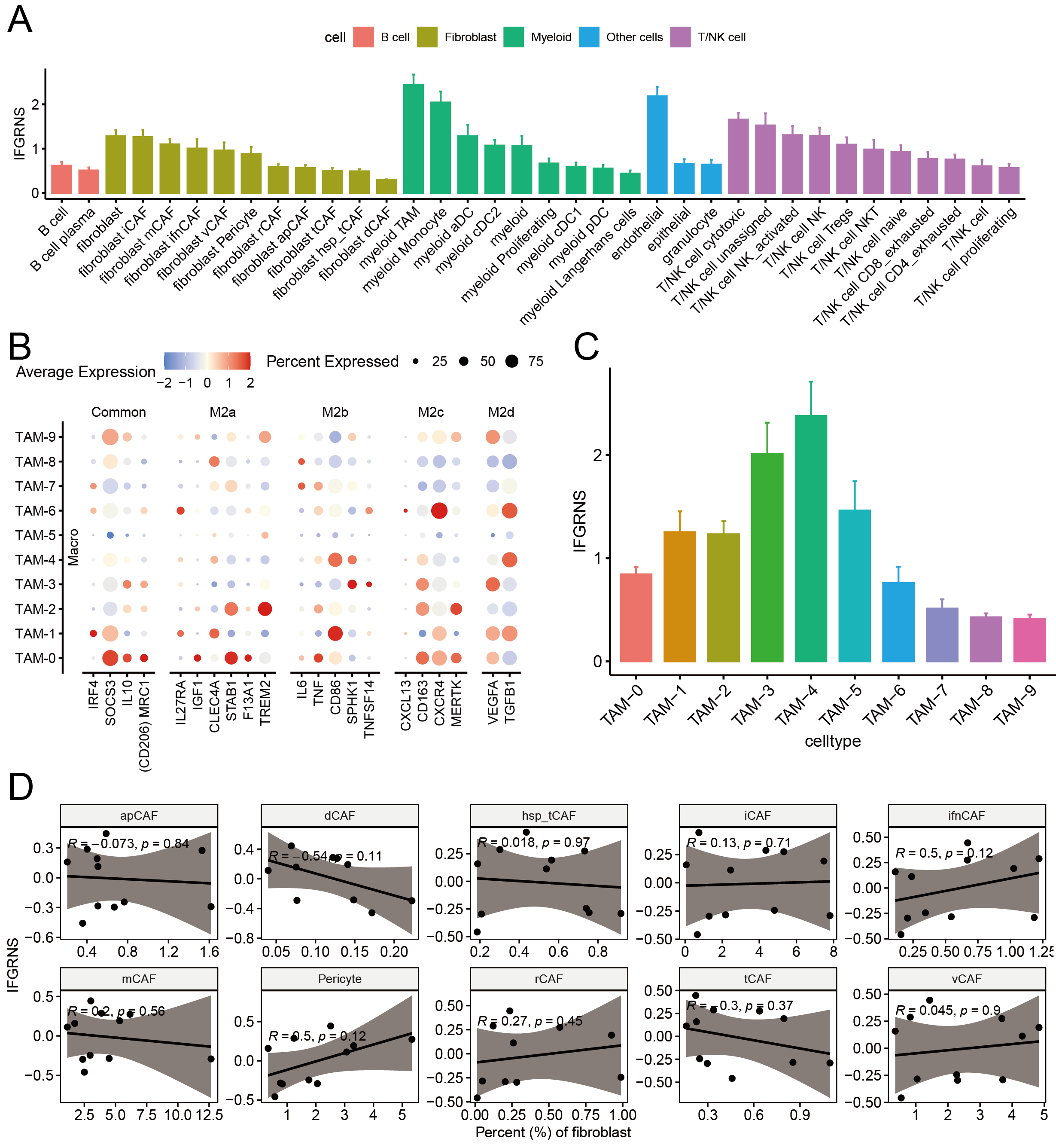


### Figure S15

**Kaplan−Meier survival curves of patients with high IFGRNS scores and low IFGRNS scores in clinical trial cohorts of anti-PD1 immunotherapy. A**, Kaplan−Meier survival curves for progression−free survival. **B**, Kaplan−Meier survival curves for overall survival. The patients in each dataset were divided into high-score and low-score group based on the given cutoff, which were the optimal cutoff and could best reflect the discrepancy between two group. The optimal cutoff and adjusted p value were calculated by function ‘cutp’ in the R package ‘survMisc’. mUC, metastatic urothelial cancer; mRCC, metastatic renal cell carcinoma; aRCC, advanced renal cell carcinoma; NSCLC, non-small-cell lung cancer; HCC, hepatocellular carcinoma; Atezo, atezolizumab; Pembro, pembrolizumab. Opdivo is also the nivolumab, and Keytruda is also the pembrolizumab.


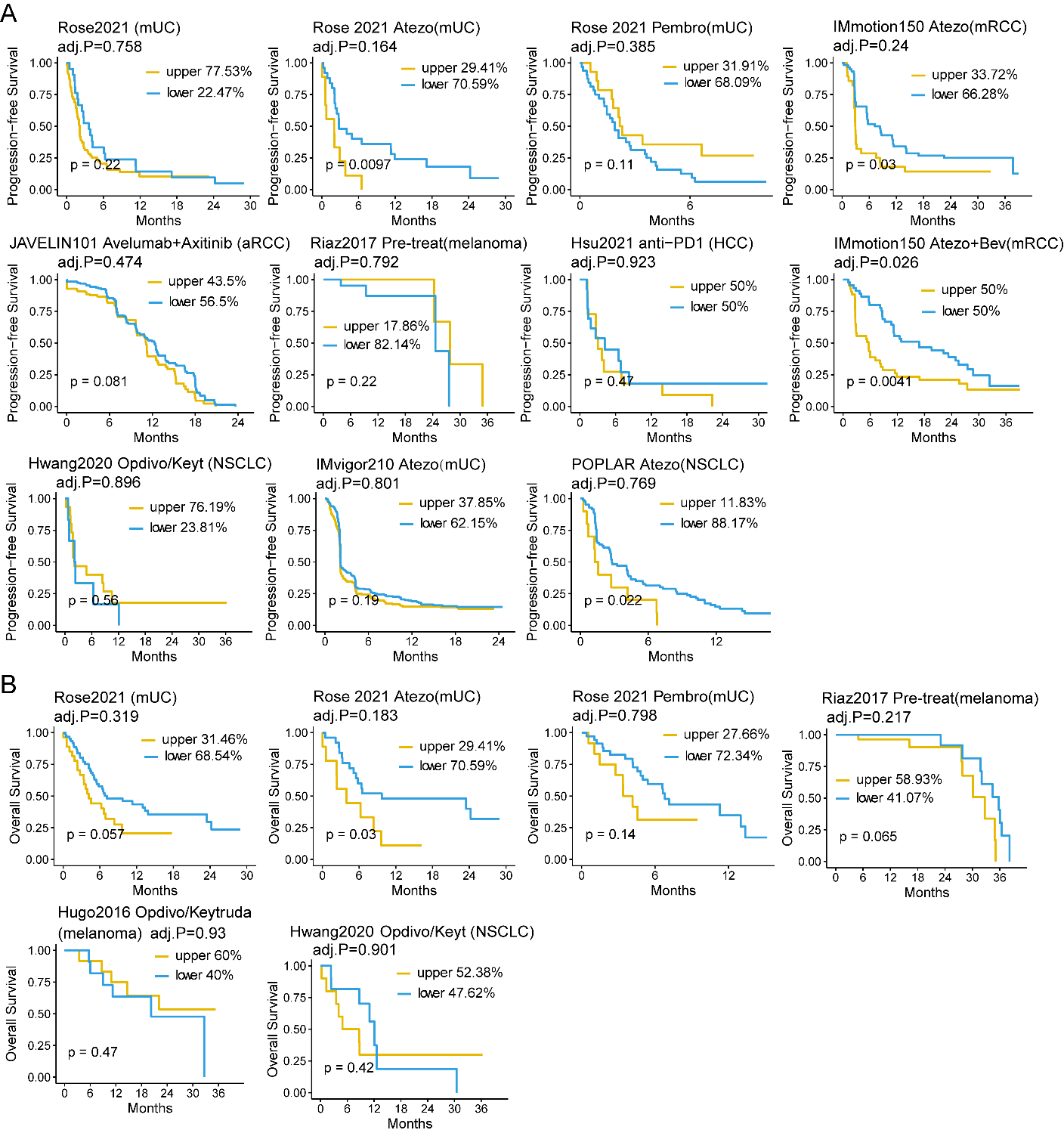


### Figure S16

**Boxplots showing the differences in IFGRNS and other anti-PD1 responsive/resistant signature scores across tumor regression response in the clinical trial cohorts with the anti-PD1 therapy.** The upper, middle, and lower hinges of the box plot are 75th, 50th, and 25th quartiles, and the whiskers extend to the range below and above, respectively. CR, complete response; PR, partial response; SD, stable disease; PD, progressive disease. mUC, metastatic uroth elial cancer; HCC, hepatocellular carcinoma; Atezo, atezolizumab; Pembro, pembrolizumab. Opdivo is also the nivolumab, and Keytruda is also the pembrolizumab.


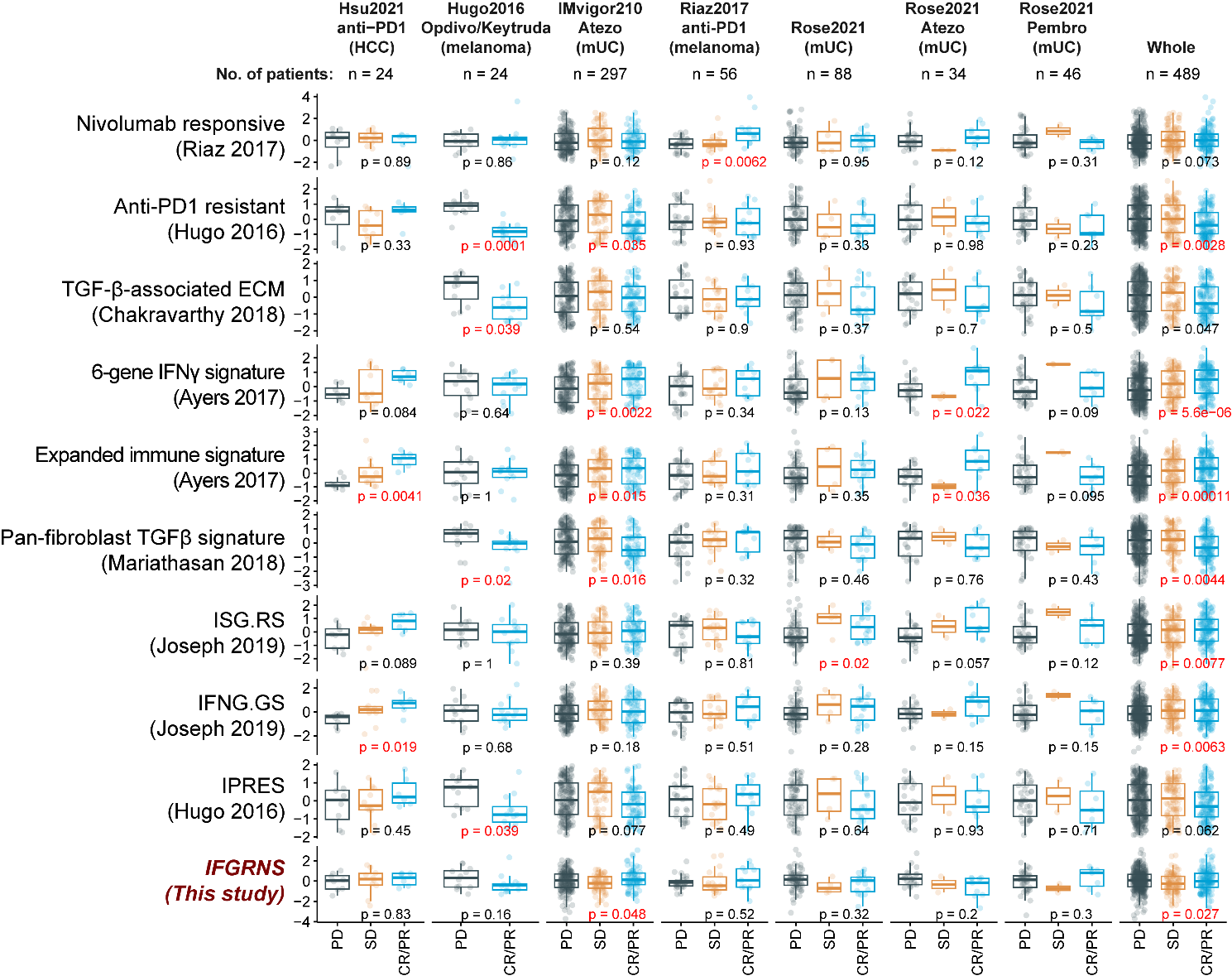


### Figure S17

**Comparison of C-index and AUC for IFGRNS and other ICI-Responsive signatures in predicting progression-free survival for patients treated with anti-PD-1 therapy.**


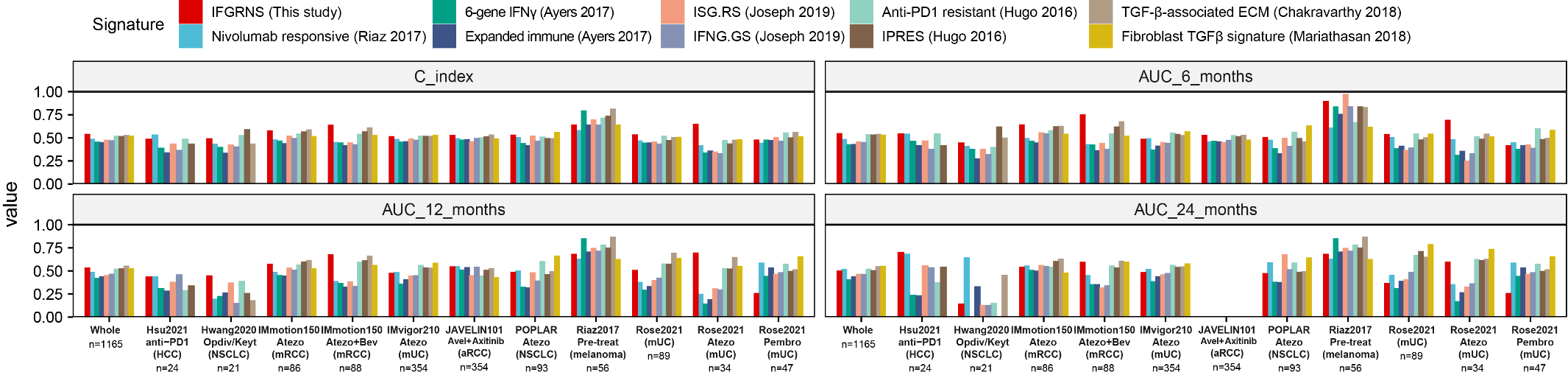


### Figure S18

**Comparison of C-index and AUC for IFGRNS and other ICI-Responsive signatures in predicting overall survival for patients treated with anti-PD-1 therapy.**


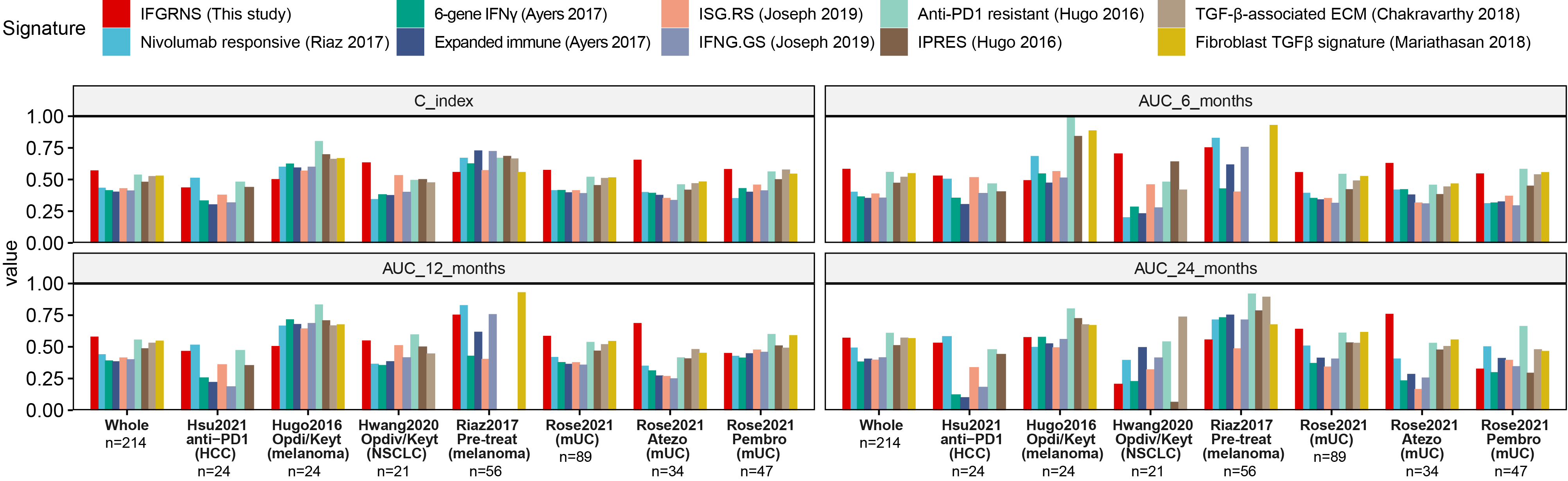


### Figure S19

**Hazard ratio and 95% confidence interval (CI) for disease-specific survival (DSS) of the IFGRNS and other anti-PD1 responsive/resistant signature scores for the training sets (red) and testing sets (black) in the cohorts of TCGA data.** The patients in each dataset were divided into high-score and low-score groups by the median of the scores. Hazard ratios were calculated with COX regression (high-score v.s. low-score groups).


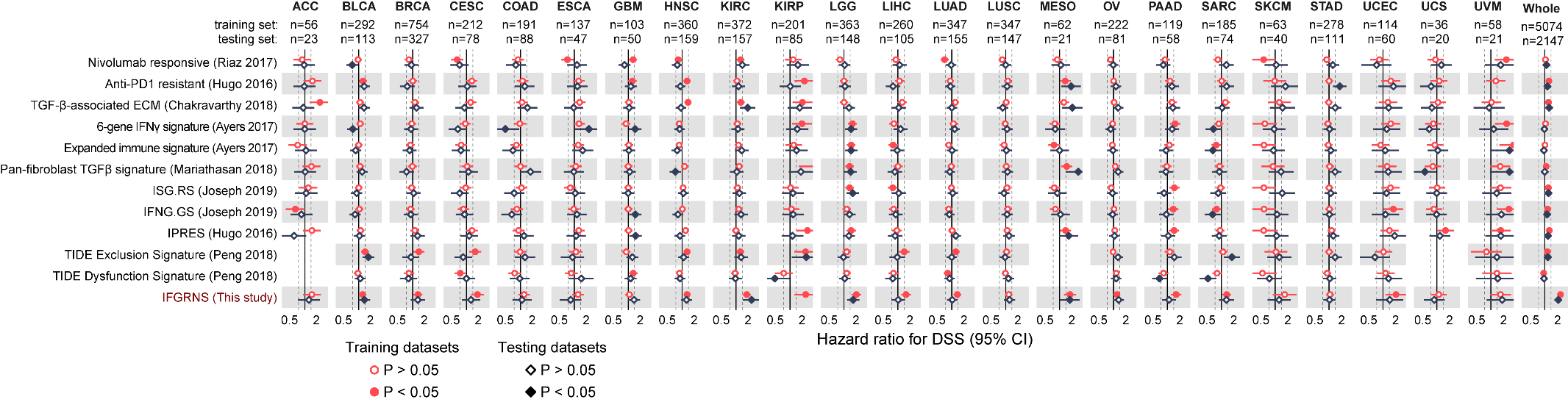


### Figure S20

**The single-cell data analysis of the murine pancreatic cancer model receiving anti-PD1 treatment. The heatmap indicating the subtypes of B cells (A), T cells (B), monocytes/DCs (C), and macrophages (D) and their marker genes for the classification.**


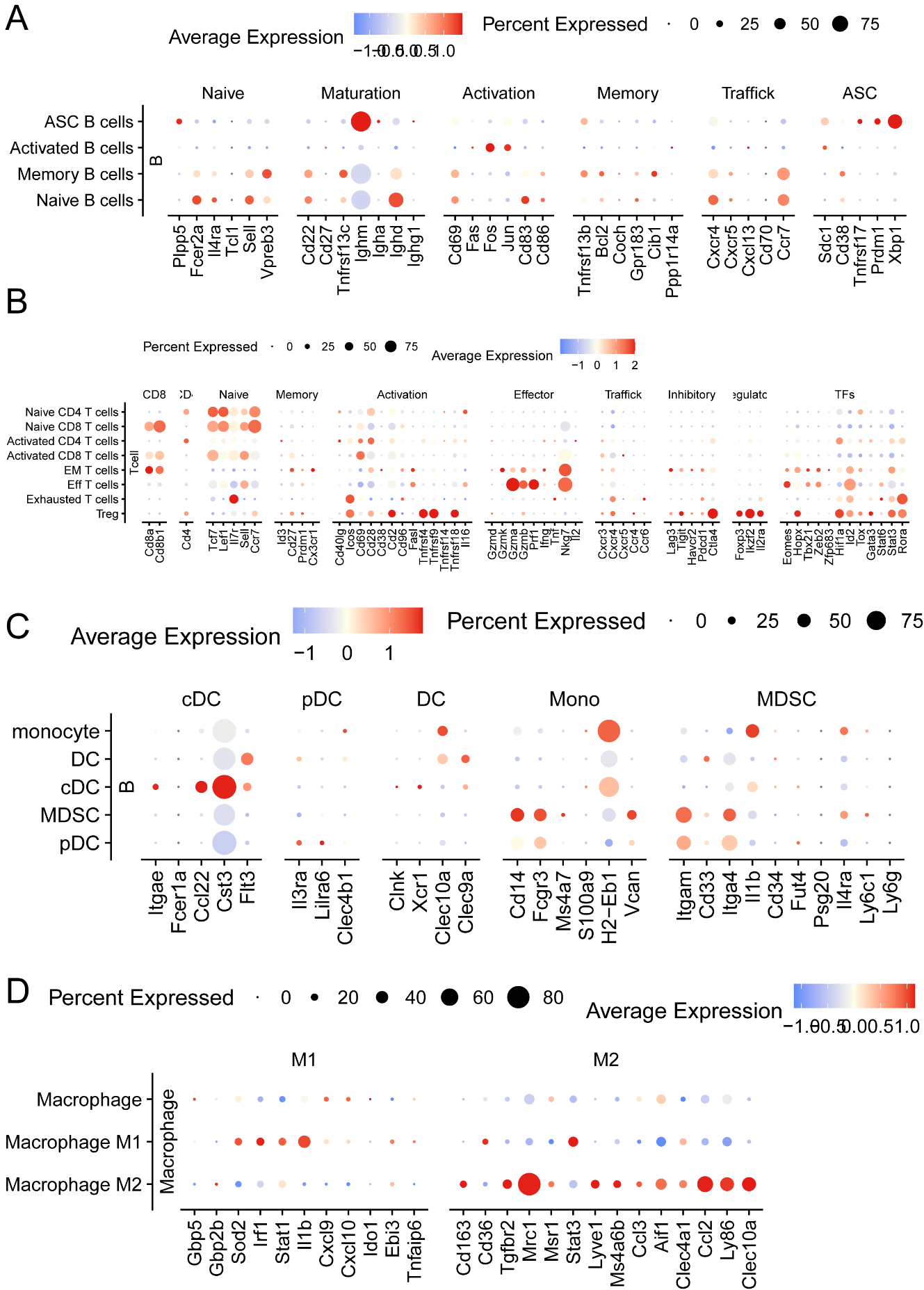


### Figure S21

**IFGRNS scores across the subclusters of the M2 macrophage in the murine pancreatic cancer receiving anti-PD1 treatment. A,** 2D UMAP visualization of all M2 macrophages and subclusters. **B**, the dot heatmap indicating the subclusters and their marker genes for the classification. **C**, a heatmap showing the expression levels of IFGRNS across various subclusters, comparing samples treated with anti-PD1 to untreated controls. Each row represents a subcluster, with expression values normalized and scaled for clear comparison. Adjacent to the heatmap, a bar summarizes the aggregate IFGRNS expression for each subcluster across all samples.


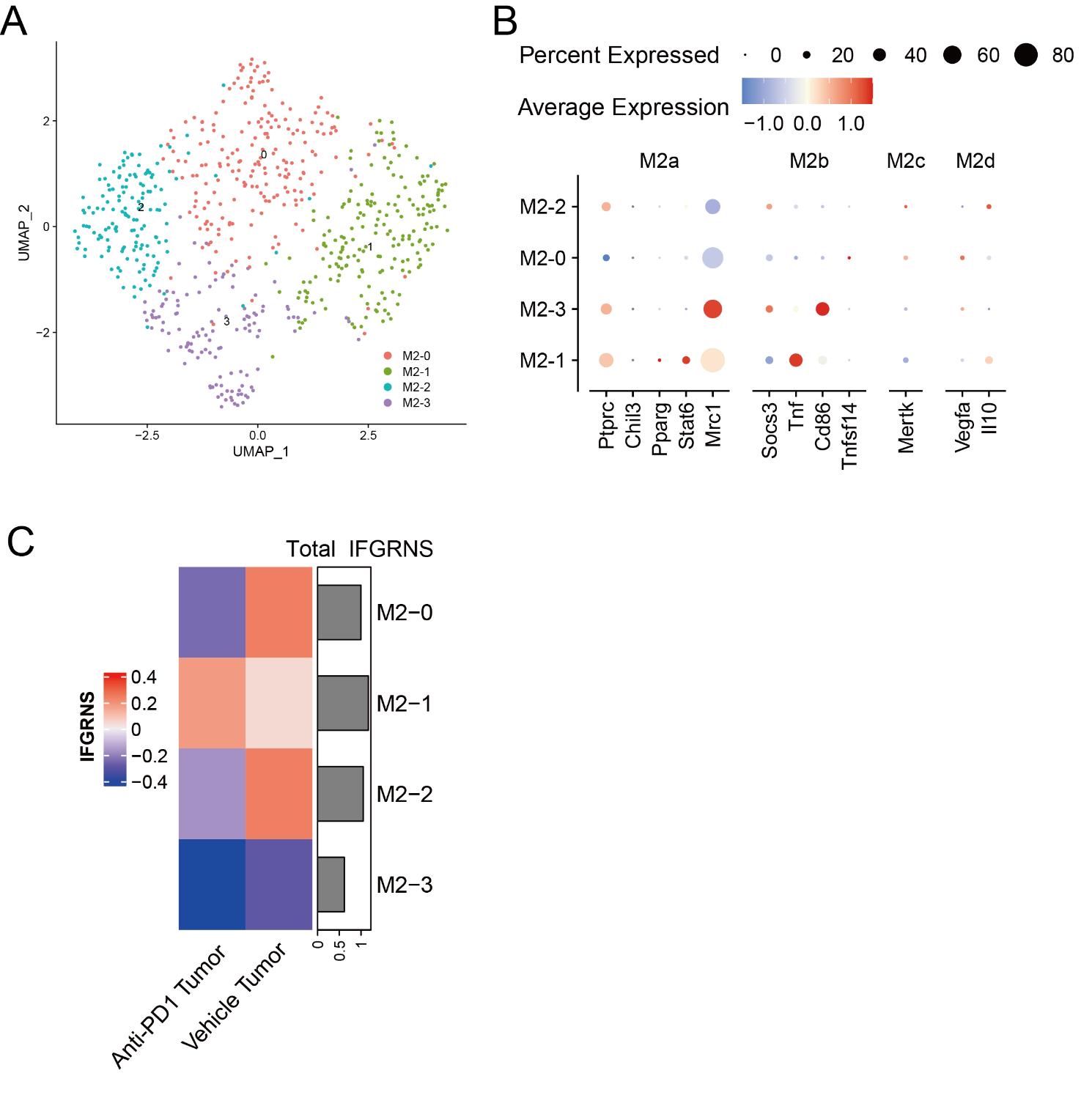

Supplement: Supplementary file 1 — Supporting Information [file CTM2-15-e70139-s001.docx]
